# Cost-Effective Sequencing of Full-Length cDNA Clones Powered by a *De Novo*-Reference Hybrid Assembly

**DOI:** 10.1371/journal.pone.0010517

**Published:** 2010-05-07

**Authors:** Reginaldo M. Kuroshu, Junichi Watanabe,, Sumio Sugano, Shinichi Morishita, Yutaka Suzuki, Masahiro Kasahara

**Affiliations:** 1 Department of Computational Biology, Graduate School of Frontier Sciences, The University of Tokyo, Kashiwa, Japan; 2 Department of Medical Genome Sciences, Graduate School of Frontier Sciences, The University of Tokyo, Tokyo, Japan; 3 Bioinformatics Research and Development (BIRD), Japan Science and Technology Agency (JST), Tokyo, Japan; Cinvestav, Mexico

## Abstract

**Background:**

Sequencing full-length cDNA clones is important to determine gene structures including alternative splice forms, and provides valuable resources for experimental analyses to reveal the biological functions of coded proteins. However, previous approaches for sequencing cDNA clones were expensive or time-consuming, and therefore, a fast and efficient sequencing approach was demanded.

**Methodology:**

We developed a program, MuSICA 2, that assembles millions of short (36-nucleotide) reads collected from a single flow cell lane of Illumina Genome Analyzer to shotgun-sequence ∼800 human full-length cDNA clones. MuSICA 2 performs a hybrid assembly in which an external *de novo* assembler is run first and the result is then improved by reference alignment of shotgun reads. We compared the MuSICA 2 assembly with 200 pooled full-length cDNA clones finished independently by the conventional primer-walking using Sanger sequencers. The exon-intron structure of the coding sequence was correct for more than 95% of the clones with coding sequence annotation when we excluded cDNA clones insufficiently represented in the shotgun library due to PCR failure (42 out of 200 clones excluded), and the nucleotide-level accuracy of coding sequences of those correct clones was over 99.99%. We also applied MuSICA 2 to full-length cDNA clones from *Toxoplasma gondii*, to confirm that its ability was competent even for non-human species.

**Conclusions:**

The entire sequencing and shotgun assembly takes less than 1 week and the consumables cost only ∼US$3 per clone, demonstrating a significant advantage over previous approaches.

## Introduction

In the last several years, the genomes of many species have been sequenced [1], [2], [3], [4], [5], [6], [7], [8], [9], [10], [11]. The identification and determination of the structure of functional elements of genomes has played a major role in supporting biological and medical research to elucidate biological processes. In this context, many approaches have focused on the investigation of transcribed regions in genomes, typically by sequencing mRNA transcripts converted into cDNAs. For functional analysis, cDNAs can also be isolated and then cloned, allowing test of gene functions and production of proteins of interest.

Recently, with the advent of second-generation sequencing instruments such as Illumina Genome Analyzer (Illumina GA) and AppliedBiosystems SOLiD (AB SOLiD), genome-wide comprehensive approaches that integrate RNA-sequencing and the second generation sequencers succeeded in capturing both expressed regions and expression levels of genes without cloning individual mRNAs [12], [13], [14], [15], [16], [17], and showed higher sensitivity and resolution than array-based systems. Detection of alternative splicing events is also an important application of these methods since the ENCyclopedia Of DNA Elements (ENCODE) pilot project revealed that on average 5.4 transcripts are expressed at one locus [24] and another report estimated that alternative splicing occurs in about 95% of multi-exon genes in major human tissues [25]. Some recent efforts using RNA-sequencing and second generation sequencers confirmed the presence of considerable amount of known splice events as well as some candidate new splice events, demonstrating its capability to detect alternative splicing events [13], [23], [25], [26], [27], [28]. However, the high complexity of transcriptome poses many difficulties in unambiguously determining the sequence and the connectivity of distant exons of individual alternative isoforms [29]. In fact, an attempt using 454-based RNA-sequencing, which generates longer reads than Illumina GA and AB SOLiD, to produce a *de novo* assembly of a non-model species transcriptome required the additional use of clone sequences, genomic data from a related model species and some human processing to reconstruct full sequences of individual isoforms in alternative splicing regions [30].

In general, sequencing full-length cDNA clones is still the most robust and accurate way to determine the exon-intron structure of a given set of genes particularly when alternative spliced isoforms possibly with different lengths are involved, because cDNA clones contain isolated transcripts so that the difficulties in the reconstruction of whole transcriptomes by non-clone-based methods can be avoided. Indeed, to this end, two groups recently reported cDNA-clone-based sequencing methods to identify alternative splicing transcripts using reverse transcription-PCR and subsequent cDNA cloning [14], [31]. Sequencing full-length cDNA clones can be the last resort if the exon-intron structure of target transcripts is difficult to determine at single base pair resolution by non-clone-based methods.

To obtain a complete sequence from a full-length cDNA clone, one usually starts by end-sequencing the cDNA clone using the Sanger method [32]. This is usually followed by either primer walking [33], which is a laborious process that requires designing thousands of custom primers, or multiclone-shotgun sequencing, which reads shotgun fragments derived from a mixture of multiple cDNA clones using the Sanger method and assembles the fragments into the original cDNA sequences separately [34], [35], [36]. Recently, this approach was extended to use Roche GS-FLX sequencer to achieve a throughput of 880 cDNA clones per run [31].

Replacing the conventional Sanger sequencer or GS-FLX sequencer with Illumina GA is a natural and cost-efficient extension to the existing approach of multiclone-shotgun sequencing of cDNA clones because Illumina GA can reduce per-base running cost by at least two orders of magnitude compared to Sanger sequencers. However, various challenges remain, due to the different characteristics of Illumina GA, such as the short-read length, higher sequencing error rate, and non-uniform shotgun-read distribution.

Assembling short reads tends to result in shorter contigs, while longer reads (i.e., Sanger reads) are more effective for avoiding ambiguity in assembly originating from repetitive sequences. Also, it remains unknown whether uniformity of a shotgun-read distribution is sufficient for short-read assembly even under modest sequence coverage. In *de novo* assembly, shorter reads require subsequent reads to appear in a short distance, demanding more uniform distribution of shotgun reads or higher sequencing coverage.

To mitigate these problems, an algorithm called “reference assembly” is often used. Reference assembly requires a standard genome that is fairly close to the genome of a given focal sample, which may not be sequenced. Input short reads are aligned against the reference genome to avoid ambiguity in contig/scaffold production. Pop et al. [37] first introduced the notion of comparative assembly, in which alignments against the reference genome were used to guide the assembly process, and many researchers are recently developing short-read reference assembly algorithms, in which short reads generated by second-generation sequencers were aligned against the reference genome and subsequently assembled [38], [39].

Previous attempts to assemble RNA-sequencing short-read data have either used *de novo* or reference assembly [27], [28], [40], [41]. Here, we introduce a new approach that integrates *de novo* and reference assembly approaches for the assembly of cDNA clones and demonstrate that the use of a reference genome sequence improved the quality of the final assembly output. For clone identification purpose, integration with Sanger reads from clone ends is also implemented.

**Figure 1:**
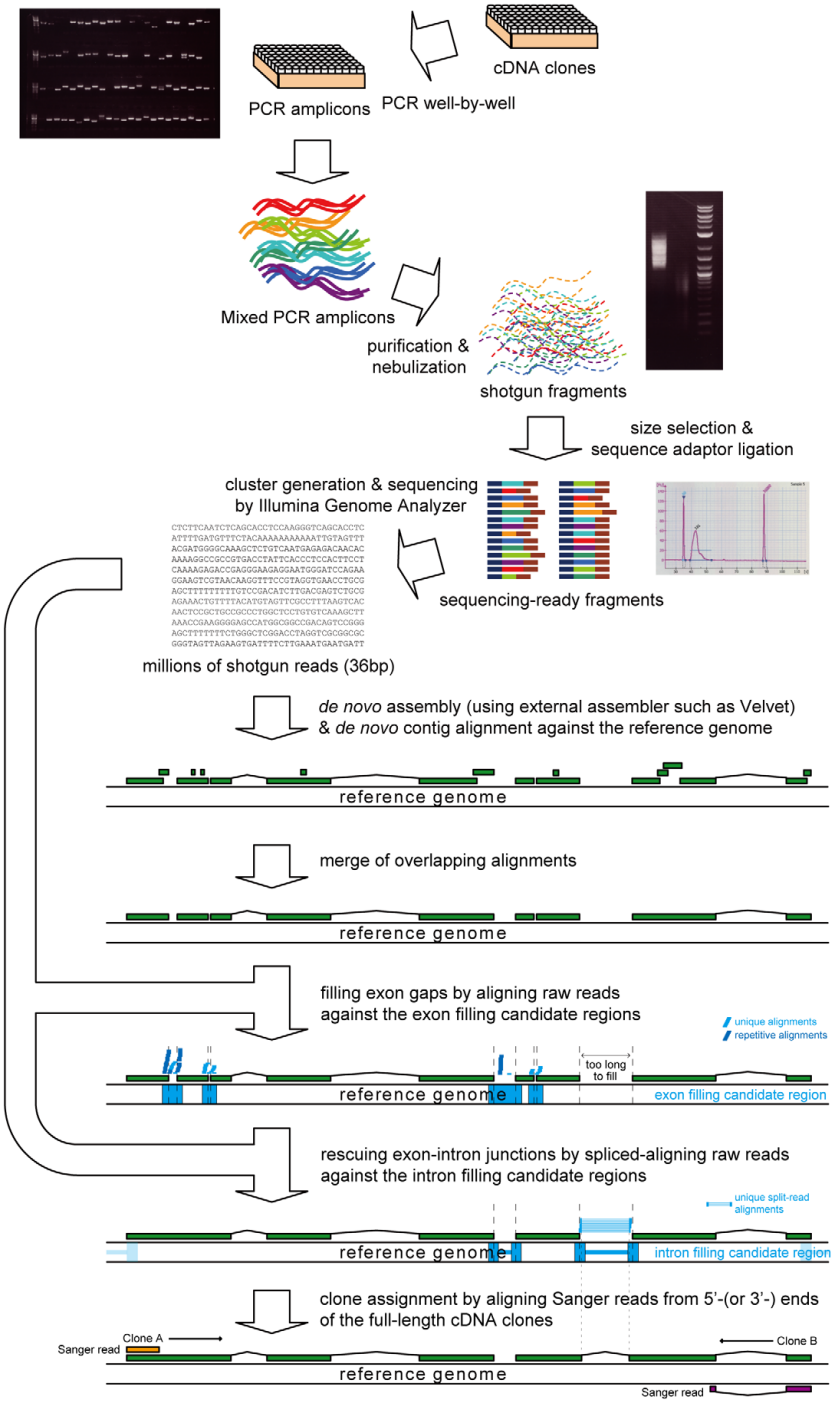
Whole full-length cDNA clone sequencing process using MuSICA 2. Target cDNA clones are prepared (for example, 800 clones per flow cell). The clones are individually amplified by PCR. The amplicons are mixed in equal volumes, and then fragmented by nebulization. The fragments of appropriate size for sequencing are size-fractionated by gel electrophoresis, followed by sequence adaptor ligation. A number of 36-bp reads are collected using the Illumina GA. The obtained reads are assembled by an external *de novo* assembler such as Velvet. The assembled contigs are then aligned against the reference genome sequence to merge overlapping contigs. Missing exons are filled by aligning raw reads against small gaps between the merged contigs. Missing introns (links between exons) are recovered by spliced alignment of the raw reads. The final contigs are associated with individual full-length cDNA clones by Sanger reads from either/both ends of the clones.

In this paper, we describe a new system called **Mu**lticlone **S**hotgun **I**ntegrated **c**DNA **A**ssembler 2 (MuSICA 2), which reconstructs full-length cDNA sequences from numerous short (36 nucleotides, nt) shotgun reads of full-length cDNA clones using an Illumina GA (Fig. 1). A set of full-length cDNA clones was prepared, and each full-length cDNA clone in the set was amplified by PCR. The mixture of equal volume of the amplified cDNA solution was nebulized to yield short fragments. After size-fractionation through gel electrophoresis, sequencing adaptors were ligated to the fragments, and millions of short single-end shotgun reads were obtained from a single flow cell lane of Illumina GA (Table S1). The collected short reads were then assembled by MuSICA 2 to output the full-length cDNA sequences accurately as possible. The assembly algorithm of MuSICA 2 is a hybrid of reference assembly and *de novo* assembly, thus benefits from advantages of the two assembly strategies, while a single strategy alone was unsatisfactory.

Direct *de novo* assembly of short reads [42], [43], [44], [45], [46], [47] at first seemed a straightforward way to reconstruct the full-length cDNA sequences. Several *de novo* assemblers were tried; however, the output contig size remained much shorter than the target full-length cDNA clones of this study (see Text S1). Meanwhile, we developed a reference genome assembly algorithm, MuSICA 1 (not described in this paper), that aligned shotgun reads against the reference genome and assembled them into contigs. Although the use of the reference genome improved the number of completely reconstructed full-length cDNA clones, we also found that the results of the reference assembly and the *de novo* assembly were complementary; a hybrid approach of *de novo* and reference assembly could alleviate issues that cannot be coped with by either approach. For example, *de novo* assembly algorithms were so conservative to avoid misassemblies that contigs were often too fragmented. Indeed, after aligning disjoint contigs against the reference genome, we found many contigs overlapping on the genome actually, allowing us to merge those overlapping contigs reliably with the help of the reference genome.

Care had to be taken to use reference assembly because it suffered from two drawbacks. The first concern was locally-intensive (e.g., more than 3 bp mutations/indels in a 36-bp read) polymorphisms between the reference genome and the target full-length cDNA clones, which made it difficult to find alignments between short reads and the reference genome. Since the strain of cDNA libraries is often different from that of the reference genome, alignment of reads against the reference genome should allow locally-intensive polymorphisms to pursue the continuity of the assembled sequences of full-length cDNA clones, but it is almost prohibitive because of the low specificity of such alignment. The second issue was how to detect exons shorter than the read length of Illumina GA. In reference assembly, spliced alignment was required to find exons shorter than 36 bp (the read length), but splicing short reads into two parts deteriorated the alignment specificity significantly, leading to substantial false-positives. We found that 14% of the clones in our human cDNA clone set had at least one exon shorter than 36 bp and that they were difficult to be assembled correctly by pure reference assembly. We concluded that reference assembly alone was likely to cause incorrect reconstruction of full-length cDNA sequences and produce many false-positive alignments. Meanwhile, *de novo* assembly does not suffer from the problem of missing short exons because it assembles the reads directly into the cDNA contigs without alignment to the reference genome.

In our hybrid approach, initial contigs are created using an externally available *de novo* assembler, and then they are improved using the reference assembly approach (Fig. 1). Disjoint *de novo* contigs that were overlapping on the reference genome in reality were detected and merged by alignment of the *de novo* contigs with the reference genome. Some partial *de novo* assembly could be fixed by alignment of the shotgun short reads to the reference genome.

## Results

### Experimental design and three sample cDNA clone sets

We prepared three input clone sets of full-length cDNA clones to accomplish the following: test the feasibility of shotgun sequencing cDNA clones using Illumina GA; investigate how multiplicity (the number of full-length cDNA clones in a clone set) affects the quality of the result, and examine whether the effectiveness and accuracy of our approach depends on a particular species. Each full-length cDNA clone set was shotgun sequenced individually using one lane of a flow cell.

To test the feasibility of shotgun sequencing cDNA clones, we randomly chose 200 non-overlapping clones from human full-length cDNA clones [33] whose complete nucleotide sequences had been sequenced independently by the conventional primer-walking method using Sanger sequencers. Non-overlapping here refers to the criterion that no two clones shared the same genomic regions, which can be easily confirmed by aligning the finished full-length cDNA sequence against the reference genome. This set of 200 cDNA clones (named “library 1”) was ideal for accuracy evaluation of MuSICA 2 because of the high sequence quality of every full-length cDNA clone achieved by the conventional primer-walking method. Fig. S1A shows the length distribution of these 200 cDNA sequences in library 1, which averaged 2,058 nt in size. One of the 200 clones was found to be chimeric by its alignment to the reference genome, and was therefore excluded from later analyses. When we selected the clones, we did not check whether the clones would share similar nucleotide sequences (e.g., repetitive sequences) that may potentially make the subsequent assembly process more complex and difficult.

To investigate how multiplicity affects the quality of the assembly result, we prepared another cDNA clone set, which we named library 1+2. It contained all the 200 full-length cDNA clones in library 1 and an additional set of 600 full-length cDNA clones to reduce the sequence coverage for the shared 200 full-length cDNA clones. The additional 600 full-length cDNA clones were chosen from existing human full-length cDNA clones [48] while avoiding any pair of clones that suggests the same transcript. The full nucleotide sequences of the additional 600 full-length cDNA clones are unknown. Library 1+2 had four times as many cDNA clones as library 1 did, so that lower sequence coverage was expected. To understand how multiplicity might affect the quality and accuracy of our approach, we compared the assembly results for library 1 and library 1+2. As the two libraries shared the 200 full-length cDNA clones, the reproducibility of our approach could be also validated by looking to what extent the shared cDNA clones were reconstructed similarly across the libraries.

To examine whether the effectiveness and accuracy of our approach depends on a particular species, we prepared library 3, a collection of 780 cDNAs from *Toxoplasma gondii*. The human genome is one of the most reliable genome sequences in terms of base quality and continuity, whereas newly analyzed genomes may not be of the same quality. Therefore, measuring the full-length cDNA reconstruction accuracy for non-human species is of great interest to suggest that our approach is applicable to a broad range of species. We expected polymorphisms of higher rate between the reference genome and the shotgun reads, and therefore the clone set was suitable for testing tolerance to polymorphisms. As a validation full-length cDNA clone set, 152 clones in library 3 were manually finished by the conventional primer-walking using Sanger sequencers (see Fig. S1B for the size distribution).

### Shotgun library preparation and sequencing

Figure 1 illustrates the whole process of our hybrid assembly approach. The detail of amplification of full-length cDNA clones and subsequent sequencing is described in Methods. One lane was used for each full-length cDNA clone set. The number of obtained raw reads, which were 36 bp in length, ranged from 4.2 million to 6.4 million. (see Text S1 for details). Reads with any ambiguous bases (e.g., N) were eliminated. For *de novo* assembly, purity filter was applied using default settings of Solexa pipeline, whereas the reference assembly process utilized non-filtered reads to obtain more aligned reads.

### Hybrid assembly approach

After millions of short shotgun reads were obtained, an external assembler was used to produce initial contigs. We used Velvet [47] as the external assembler because it gave the best continuity among tested *de novo* assemblers (see Text S1), though any other improved assembler can be used instead in the future.

As our hybrid assembly greatly relies on the quality of initial contigs produced by *de novo* assembly, a good parameter selection for Velvet is a major determinant in our approach. Among a variety of parameters, hash length and read length, are particularly important, and therefore, Velvet was run with hash length ranging from 19 bp to 29 bp and read length from 26 bp to 36 bp, after which the initial contig set was selected among the assemblies with different read length and hash length parameters. We found that the N50 contig lengths produced in *de novo* assemblies were highly correlated with the accuracy of the assembly result produced by MuSICA 2 (Fig. S2); hence we used the Velvet assembly with the best N50 contig length. The read lengths that produced the best *de novo* assembly were 36 bp, 36 bp and 34 bp, for library 1, 1+2 and 3, and the hash lengths selected were 27 bp, 23 bp and 21 bp, for library 1, 1+2 and 3, respectively.

The contigs produced by the external assembler were then aligned against the reference genome using the BLAST-like Alignment Tool (BLAT) [49]. The alignment with the best alignment score was kept for each contig. When multiple hits tied in alignment score, they were all discarded as repetitive hits. Contigs overlapping on the genome are then merged (see Methods), reducing the number of contigs by more than 30% (Table 1).

**Table 1:** Hybrid assembly statistics for individual libraries.

	Library 1		Library 1+2		Library 3	
Full-length cDNA clones	200		800		780	
	# contigs	N50 (bp)	# contigs	N50 (bp)	# contigs	N50 (bp)
*de novo* contigs	424	1,721	2,824	1,214	1,910	1,184
After merging overlapping contigs	283	1,912	1,605	1,558	965	1,542
After exon gaps are closed	194	2,157	1,019	1,770	709	1,707
After intron gaps are closed	187	2,157	890	1,864	674	1,741
After association with clones	161	2,116	695	1,925	679	1,764

The number of contigs is presented for each step in the assembly process. Ideally, the figures converge to the number of PCR-amplified full-length cDNA clones in each library as it goes down the process. However, the actual number of PCR-amplified full-length cDNA clones is not known except for library 1 (158 clones). Less-amplified clones were often reconstructed well but their bands in a gel electrophoresis picture were too faint to identify, making it difficult to experimentally measure the number of PCR-amplified full-length cDNA clones without aligning the shotgun reads. In the last step, the N50 contig size for library 1 decreased because some contigs from the previous step were not associated with any Sanger read and were then discarded to avoid false positives. For library 3, the number of contigs increased in the last step because multiple Sanger reads were associated to the same contig, which was possibly an error that could be eliminated if the output was manually examined.

The benefit of this contig improvement by reference alignment over *de novo* assembly is four fold: (a) *de novo* assemblers often yield short contigs due to its inherent difficulty, but reference alignment allows us to merge contigs according to their ranges on the genome sequence; (b) not all of the contigs are correctly assembled, but misassembled contigs are corrected simply by discarding unaligned parts; (c) even repetitive short (36-mer) regions can be reliably covered when a contig that covers them contains at least one unique region that aligns to only one location on the genome; (d) contigs are usually longer than reads, so that spliced alignment often retains sufficient specificity even when the length of exons are shorter than reads.

After merging overlapping contigs, the merged contigs do not always represent complete full-length cDNA sequences presumably because those contigs are fragmented due to lack of sequence coverage. To check this hypothetical reason, we aligned raw shotgun reads against the finished full-length cDNA clones, and we found that entire clones were usually covered by the raw shotgun reads, with the exception that regions towards the ends of the clones were underrepresented in the shotgun reads (see Supporting Information). Some parts of exons or intron were not covered by any *de novo* contigs presumably because short read *de novo* assemblers discard contigs shorter than a threshold.

To compensate for missing contigs, MuSICA 2 uses raw shotgun reads. If two contigs aligned adjacent on the genome are within 1,000 bp, which is the user-configurable parameter, the gap between them is considered for *exon filling* and is called an *exon gap candidate*. Exon gap candidates are attempted to be filled with aligned raw shotgun reads using a custom Perl script and BLAT with a permissive parameter (see Methods), allowing up to three mismatches/indels. When any single nucleotide in an exon gap candidate is not covered by any raw read, the exon gap candidate is not filled. To avoid filling repetitive regions with spurious matches, the gap is filled only when the number of unique alignments per base is greater than or equal to 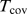 reads. 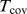 is empirically set to 2 by default, and therefore higher value might be used for higher sequence coverage (e.g., >300×). This exon filling process was capable of merging fragmented contigs, thereby reducing the number of contigs by 27–37% (Table 1).

After exon filling, MuSICA 2 tries to compensate for missing splicing junctions that *de novo* contigs fail to cover. If the distance between two adjacent contigs aligned on the reference genome is within 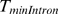 to 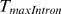, the gap between them is considered as an *intron gap candidate*. Raw reads are split into two parts, and then they are aligned against regions near both ends of the intron gap candidates to find actual introns (for spliced alignment, see Methods). At this stage, exons are expected to be nearly perfect. Therefore, the total length of the alignment targets is at least four orders of magnitudes shorter than the whole genome in the case of human (Table S2). The narrow alignment targets enabled more reads to be aligned uniquely.

### Clone name assignment

Associating individual full-length cDNA clones with the output contigs is a crucial step to identify the clone material corresponding to any user-requested contig for wet-lab experiments. To this end, we collected Sanger reads from both ends of the full-length cDNA clones as input, though they were not incorporated into the contig generation except for cases explained below. A Sanger read from either end of a clone will be sufficient for clone name assignment.

We aligned the input Sanger reads against the output contigs produced by MuSICA 2 using BLAT. Alignments shorter than 30 bp or those with identity less then 90% were discarded. The filtered alignments usually associated each clone with one contig. Conflicting alignments were resolved on a best-match basis when a single clone could be associated with multiple contigs aligned on totally different locations on the genome. When the 5′-end of a Sanger read did not align well to the associated contig, the unaligned portion of the Sanger read was added to the contig (Fig. S3). This occurs when the sequence coverage near the ends of the clone was so low that the contig was truncated. 57 (lib. 1) and 460 (lib. 1+2) full-length cDNA clones were improved through this process, and the ratio of the output nucleotides derived from the Sanger reads were 2.1% (lib. 1) and 4.3% (lib. 1+2), respectively.

### Accuracy evaluation

Library 1 consisted of 199 reference human full-length cDNA clones whose complete nucleotide sequences were previously finished by the conventional primer-walking method using Sanger sequencers. We compared the assembly results with those finished sequences to evaluate our hybrid assembly approach. We aligned the finished sequences against the reference genome to determine their exon-intron structure using BLAT. The alignment results of the finished sequences by the conventional primer-walking method were examined and corrected manually if needed (see Text S1). This structure was used as a reference exon-intron structure.

To estimate the accuracy of the assembly results produced by MuSICA 2, we directly compared the MuSICA 2 assembly with the reference clones first. The comparison suggested that the 5′- and 3′-ends of the full-length cDNA clones are difficult to determine at a base pair level due to shallow sequence coverage near the ends of the full-length cDNA clones (Fig. S4). This is because the standard Illumina nebulization protocol is inefficient in shearing DNA molecules near the ends (see Text S1). Obviously, using non-PCR methods such as plasmid extraction or TempliPhi for clone amplification would alleviate the problem because they virtually eliminate all clone ends. Although we are improving the experimental protocol, we describe the performance with the original protocol in this paper. Taking this into account, we instead focused on the consistency of the coding sequence (CDS) structure, which is a set of genomic regions that correspond to the CDS. The CDS structure comprises of a series of exon coordinates on the reference genome, and does not include any nucleotide sequences. We compared the output exon-intron structure from library 1 and library 1+2 with the reference CDS structure. The annotation of CDS for those clones was downloaded from GenBank. Of all 199 reference full-length cDNA clones, we observed that 158 clones in library 1 had at least one associated contig, whereas the other clones were not associated with any contig. The number is the same for library 1+2. We defined the other clones as “clones not amplified by PCR” because they missed shotgun sequence coverage due to failure in PCR. One may suspect that PCR failure rate was correlated with clone length, but we found no statistically significant correlation (p = 0.38 for Mann-Whitney U-test; p = 0.80 for Kormogorov-Smirnov test; see Text S1; Fig. S5).

**Figure 2:**
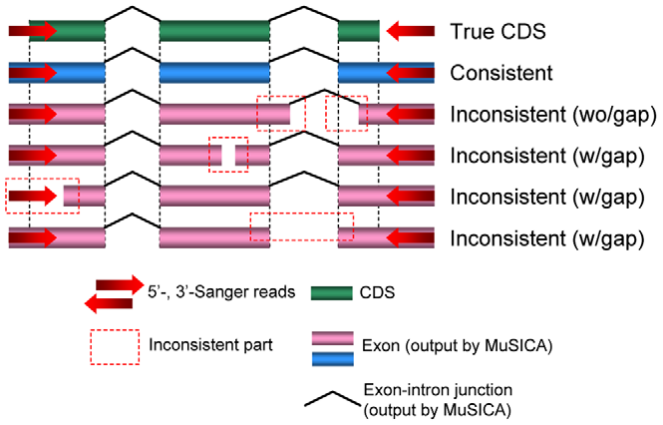
The schematic picture of CDS consistency. The reconstructed full-length cDNA is *consistent* with the reference gene if the exon-intron structure of the true CDS was completely contained in the exon-intron structure of the reference gene. *Inconsistency* was caused by different exon boundaries, sequence gaps or missing links between exons (i.e., introns). Sequence gaps were categorized into three types: (1) a gap in the middle of exons, (2) the 5′-end (or 3′-end) of the cDNA clone was missing, or (3) a link between two adjacent exons was missing. Note that in the case (3) we cannot exclude a possibility that some exon(s) between them is missing; therefore, the output transcript needs manual finishing in such a case.

**Figure 3:**
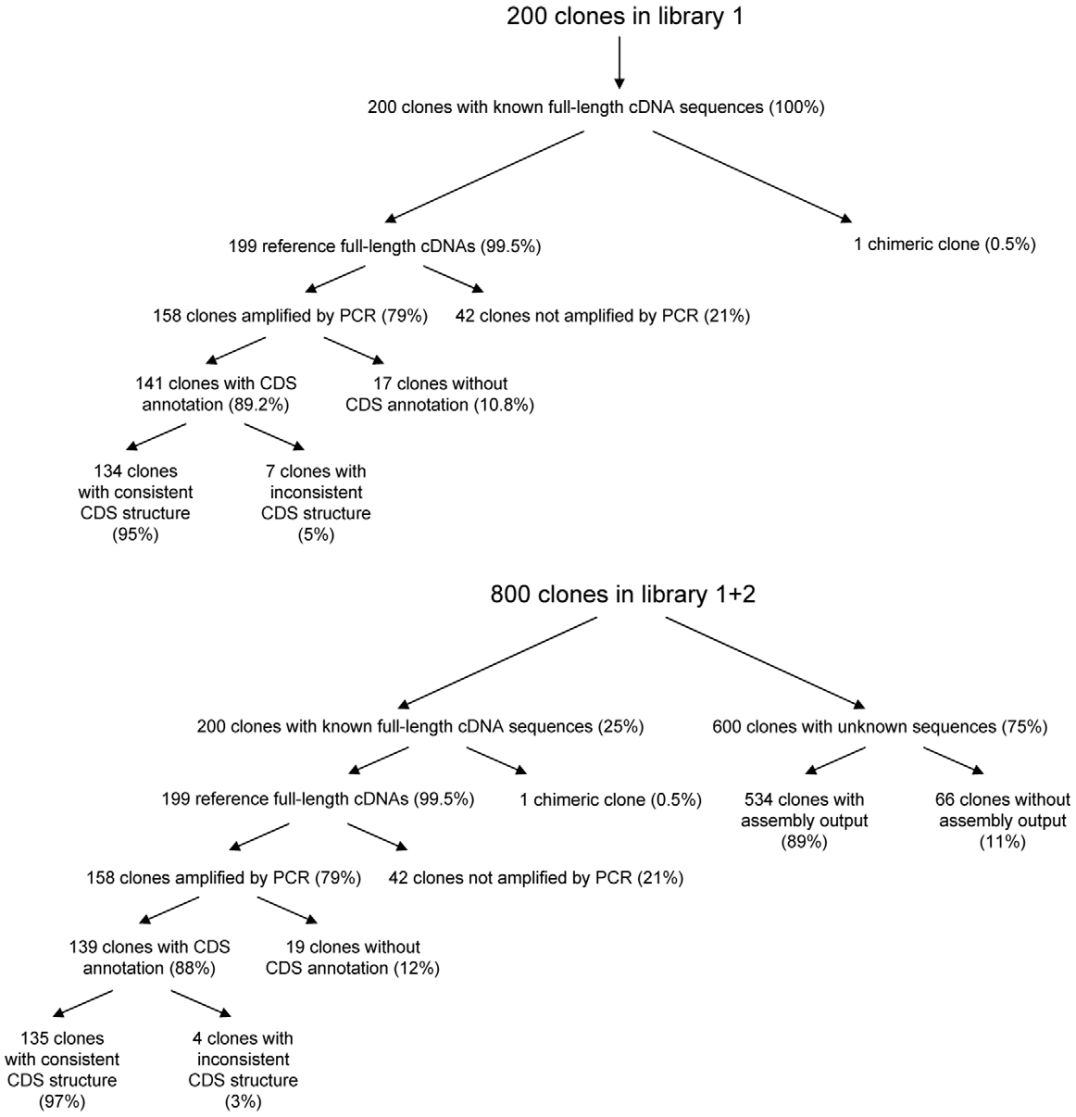
The clone fate for library 1 and 1+2. The diagram shows the accuracy evaluation process for the 200 human full-length cDNA clones in library 1 as well as the 800 human full-length cDNA clones in library 1+2. For library 1+2, the contigs associated to the clones with unknown sequences had an average length of 1,716 bp.

Table 2 compared the output cDNA sequences and the reference cDNAs in terms of the consistency of the CDS structure, which was obtained by alignment against the reference genome. Of all clones with CDS annotation and successful PCR amplification, the reference CDS structure was completely contained in the reconstructed exon-intron structure (see Fig. 2) in 95.0% and 97.1% (Table 2, Fig. 3) of the clones for library 1 and 1+2, respectively. Comparing with other approaches, MuSICA 2 produced more consistent outputs than *de novo*-based approaches improved by the use of Sanger reads (Fig. S6).

**Table 2:** Assembly statistics and accuracy evaluation for human full-length cDNA clones.

		Library 1	Library 1+2
Reference full-length cDNA clones	(A)	199	199
Reference clones amplified by PCR	(B)	158	158
%	(B/A)	79.4%	79.4%
Clones with CDS annotation	(C)	141	139
%	(C/B)	89.2%	88.0%
Clones with consistent CDS structure	(D)	134	135
%	(D/C)	95.0%	97.1%
Clones with inconsistent CDS structure	(E)	7	4
%	(E/C)	5.0%	2.9%

**A:** The number of the reference full-length cDNA clones used in the experiment. the original number was 200, but one of them was excluded due to chimerism; **B:** the number of reference full-length cDNA clones successfully amplified by PCR. Note that it does not include non-reference full-length cDNA clones; **C:** the number of successfully amplified reference full-length cDNA clones with CDS annotation. Non-reference and/or non-amplified full-length cDNA clones were excluded; **D:** the number of full-length cDNA clones that were reconstructed consistently in terms of CDS structure. See Fig. 2 for CDS structure consistency; **E:** the number of full-length cDNA clones that were not reconstructed consistently with regard to CDS structure. This always equals to C–D.

Since the CDS structure analysis does not evaluate the nucleotide accuracy, we aligned the assembly results against the reference full-length cDNA clones to evaluate the base accuracy of the assembly results (Table 3). The overall base accuracy was 98–99%; however, mismatches and indels appeared locally; the nucleotides near the contig ends were less accurate. To illustrate this, we aligned the CDS-consistent clones in the assembly results against the CDS of the reference full-length cDNA clones. The match ratio was over 99.99% (Table 4, S3 and S4), demonstrating very high base accuracy. These results suggest that the reconstructed cDNA sequences were reliable enough to annotate CDSs on the reference genome.

**Table 3:** Base accuracy of MuSICA 2 assembly.

		Library 1	Library 1+2
Total reference full-length cDNA length	(A)	325,967	328,279
Total aligned output cDNA length	(B)	317,709	320,850
Total matches	(C)	315,861	317,168
Total mismatches	(D)	71	67
Total insertions	(E)	245	269
Total deletions	(F)	1,458	1,594
Match ratio	(C/B)	99.42%	98.85%

**A:** The total number of base pairs in the reference full-length cDNA clones. Note that non-reference clones are excluded; **B:** the total number of base pairs in the output contigs; **C:** the number of base pairs matched to the reference full-length cDNA clones in the alignments (B); **D:** the number of base pairs mismatched to the reference full-length cDNA clones in the alignments (B); **E:** the number of base pairs inserted (e.g., a reconstructed contig is longer than the reference clone) in the alignments (B); **F:** the number of base pairs deleted in the alignments (B).

**Table 4:** Base accuracy of consistent coding sequences.

		Library 1	Library 1+2
Total CDS length in the PCR-amplified reference full-length cDNA clones	(A)	132405	133468
Matches	(B)	132399	133465
Mismatches	(C)	6	3
Indels	(D)	1	0
Match ratio	(B/A)	99.9955%	99.9978%

**A**: The total number of base pairs in the CDS of the reference full-length cDNA clones successfully reconstructed with regard to CDS structure. Note that library 1 and 1+2 have different sets of PCR-amplified reference full-length cDNA clones; **B:** the number of matched base pairs; **C:** the number of mismatched base pairs; **D:** the number of indels (insertions/deletions).

The accuracy evaluation for the 600 clones (library 2) that were not shared with library 1 was difficult. For example, their full-sequences are not available and therefore the correct CDSs are unknown. However, the performance seemed comparable in that the N50 contig lengths did not differ much between library 1 and 1+2 (Table 1) and that 89% of the clones had associated contigs (Fig. 3), suggesting that their accuracy and quality did not differ much from the shared 199 clones, for which we evaluated the CDS structure and the nucleotide accuracy.

### Sequence coverage analysis

Sequence coverage across clones is a factor that may affect the sensitivity and accuracy of assembly methods considerably. To assess the relationship between the accuracy of the MuSICA 2 assembly output and the sequence coverage, we produced simulated shotgun reads of reduced sequence coverage by random sampling of the raw reads. For library 1 and 1+2, simulated datasets with 10 to 100% of the reads were created and assembled by MuSICA 2. The CDS structure consistency shown in Table 5 suggested a slight tendency that deeper sequence coverage led to higher consistency, but the CDS consistency seemed to be almost saturated when the sequence coverage was over 40%.

**Table 5:** Relationship between CDS consistency and the sequence coverage.

	Coverage	10%	20%	30%	40%	50%	60%	70%	80%	90%	100%
Library 1	# consistent	117	122	125	131	129	130	128	138	127	134
	# inconsistent	16	18	14	9	10	10	12	4	11	7
Library 1+2	# consistent	105	117	118	126	128	129	130	129	133	134
	# inconsistent	23	19	21	14	11	8	8	10	6	5

Shotgun reads in library 1 and 1+2 were randomly sampled to simulate datasets of different sequence coverage. Each simulated dataset was then assembled using MuSICA 2, and the CDS structure consistency for the 199 reference full-length cDNA clones was evaluated. Full data corresponds to 100% sequence coverage. The definition of CDS structure consistency is exactly same as in Table 2. Note that the purity filtered reads, not the raw reads, were used for input to MuSICA 2, which accounts for a slight difference between Table 2 and the number of CDS-consistent/inconsistent clones at 100% sequence coverage.

**Figure 4:**
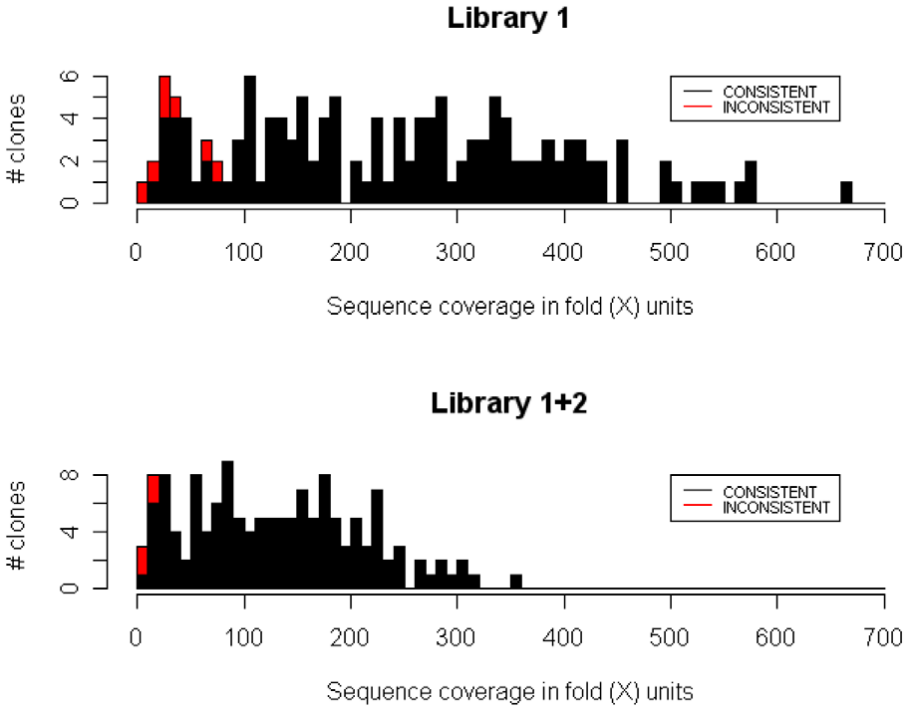
A histogram showing relationship between the sequence coverage for each clone and their assembly result classifications. The histograms show the sequence coverage distribution of clones in Library 1 and 1+2. First, we aligned the shotgun reads against the finished reference clone sequences, allowing up to 3 mismatches. Next we calculated the sequence coverage (X-axis) for each clone as follows: (# of reads aligned with the clone)×(read length (36 bp))/(the length of the clone). Y-axis shows the number of clones. Every clone is colored according to its consistency of the CDS structure; a red bar shows the proportion of the inconsistent clones in that range. Clones that were not successfully amplified by PCR are not shown in the histogram, as they always had little sequence coverage by definition. Most of the clones classified as inconsistent had sequence coverage lower than 50×, suggesting that inconsistency might have arisen due to lower sequence coverage.

**Figure 5:**
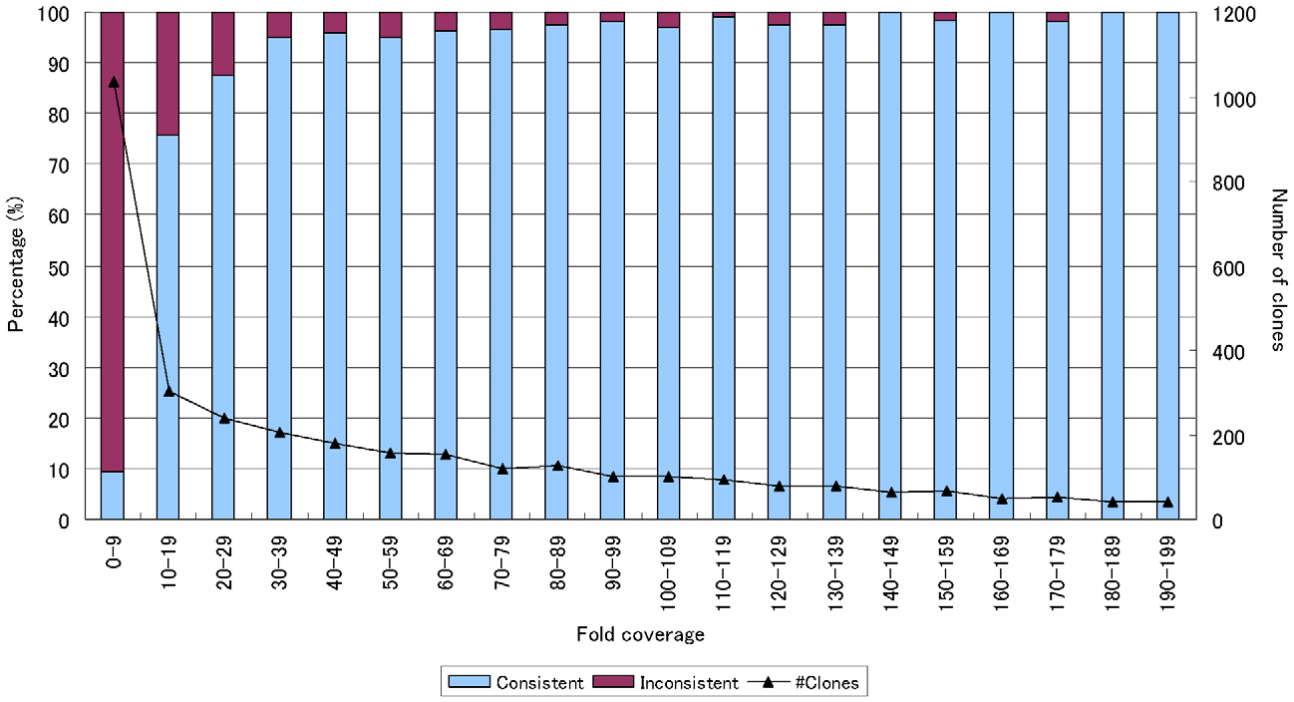
Relationship between the sequence coverage for each clone and the reconstruction accuracy in terms of CDS structure consistency. Clones in the simulated datasets were binned according to their per-clone sequence coverage. The bins were of every 10-fold. Note that every full-length cDNA clone was counted 10 times as it appeared with 10 different sequence coverage. We calculated the percentage of clones (left Y-axis) classified as consistent (or inconsistent) in terms of CDS structure for each bin. The number of clones in each bin is also shown (right Y-axis). Per-clone sequence coverage of 30-fold was sufficient to produce a CDS-consistent assembly in 95% of the cases, showing that uniform coverage distribution is highly desirable to achieve better efficiency.

This observation is somewhat different from our expectation, and we analyzed this more precisely. We found that the sequence coverage for individual clones varied widely, and therefore high (e.g., 100×) overall sequence coverage did not always warrant high sequence coverage per individual clone. Indeed, Figure 4 shows a clear tendency that clones of low per-clone sequence coverage were often not reconstructed correctly. To illustrate this more clearly, we binned the clones in all the simulated datasets by their per-clone sequence coverage (Fig. 5), confirming that sequence coverage of 30× was sufficient to correctly determine the CDS structure at an accuracy of 95%. This suggests that our hybrid assembly approach will work with higher multiplicity and can reduce sequencing costs further if more uniform sequence coverage across clones is achieved.

### Application to non-human species

We also studied the CDS reconstruction accuracy for non-human species using library 3, which consisted of 780 full-length cDNA clones from *Toxoplasma gondii*, a human pathogen. To create the reference CDS structure for accuracy evaluation, we finished 152 full-length cDNA clones in the library by the conventional primer-walking method using Sanger sequencers. Since automated annotation using homology search is less effective in identifying CDS for cDNA of *Toxoplasma gondii*, which is rather distant from the nearest sequenced species with abundant CDS annotation, we used the longest ORF as a (putative) reference CDS. Of all the 152 reference clones, 131 full-length cDNA clones were successfully amplified by PCR (Fig. S7); those clones were used as a reference in the subsequent evaluation of the CDS structure consistency.

We compared the output contigs with the reference exon-intron structure as in the case of library 1 and 1+2, and found that the CDS structure of more than 97% of the output contigs were consistent with the reference CDS structure (Table 6). This figure is similar to that for human full-length cDNAs, demonstrating the independency of our approach with regard to species or the quality of the reference genome sequence. The other statistics in Table 1 and 2 generally agree with the experiment with the human full-length cDNAs, suggesting that our approach is also effective for annotating the CDS structure of *Toxoplasma* and potentially other non-human species.

**Table 6:** Assembly statistics and accuracy evaluation for *Toxoplasma gondii* full-length cDNA clones.

		Library 3
Reference full-length cDNA clones	(A)	152
Reference clones amplified by PCR	(B)	133
%	(B/A)	87.5%
Clones with CDS annotation	(C)	131
%	(C/B)	98.5%
Clones with consistent CDS structure	(D)	128
%	(D/C)	97.7%
Clones with inconsistent CDS structure	(E)	3
%	(E/C)	2.3%

The definitions of the columns are exactly the same as in Table 2.

## Discussion

Full-length cDNA clone collections with complete sequences are valuable resources to accurately determine the exon-intron structure of a given set of genes particularly when alternative spliced forms and/or lowly expressed genes are difficult to reconstruct at base pair level by RNA-sequencing and second generation sequencers. They are often used for functional analysis of genes. To sequence cDNA clones, several methods are proposed but our approach is outstanding in low sequencing cost. Approximate cost (for consumables) per full-length cDNA clone is US$ 2.70. The precise calculation is:

US$ 3,000 (Illumina GA 1 run [50])/7 lanes (1 flowcell has 8 lanes; use 1 lane for calibration)/800 clones (per lane)/0.79 (PCR efficiency)/0.97 (accuracy)+US$ 2.00 (one-pass Sanger sequencing for both ends of the clones) = US$ 2.70.

On the other hand, previous sequencing approaches using Sanger methods are obviously much more costly. For example, Hokkaido System Science Co., Ltd. and Eurofins MWG Operon Inc. provide commercial cDNA clone sequencing service using primer-walking strategy at the price of US$ 780 and US$ 600, respectively (list price; see http://www.hssnet.co.jp/e/2/2_3_1_2.html and http://www.operon.com/products/sequencing/sequencing_pricelist.aspx; as of 2009 Sept 15), which are far more expensive than our method even considering that labor costs are included in the prices. Multiclone shotgun sequencing using the Sanger method is cheaper than primer-walking but still much more expensive than our method. Assuming 6.7× sequencing coverage and 0.81 custom primers per kilobase [36], a rough estimate of the minimum cost is:

2.0 kb (clone length)×6.7 (sequencing coverage)/800 bp (average read length)×US$ 1.00 (cost per read)+2.0 kb (clone length)×0.81 (primers per kb)×US$ 5.00 (custom primer sequencing) = US$ 24.85.

Second-generation sequencers deliver DNA sequencing at a lower cost. For example, Salehi-Ashtiani et al. [31] sequenced 820 open reading frames (ORFs) at a single run of Roche 454 FLX sequencer using shotgun sequencing; a rough estimate of the minimum consumable cost is US$ 8,500 [50]/820 (# of ORFs)+US$ 1.00 (at least one end of each clone must be sequenced by the Sanger method to associate assembled sequences with the physical clones) = US$ 11.37. This approach also provides a fast way for sequencing full-length cDNA clones but still more expensive than our method.

Since our approach has significantly reduced sequencing cost, MuSICA 2 accelerates numerous full-length cDNA sequencing projects for a variety of species, and will provide more accurate knowledge about transcriptome complexity. The collections of full-length cDNA clones are also useful for experimental analysis to reveal the biological functions of proteins. For example, recombinant proteins can be produced using expression vectors made from individual full-length cDNA clones with the complete sequences of their CDSs. Therefore, we contribute a new method for transcriptome studies that simplifies the task of assembling targeted isoforms.

Although the requirement of having Sanger reads adds extra costs beyond that of the Illumina GA sequencing reagents, to the best of our knowledge, there is no cheaper method that allows one to determine the correspondence between reconstructed cDNA sequences and individual clones, suggesting that multi-clone shotgun sequencing using the Illumina GA with additional Sanger reads is the most cost-effective method for obtaining complete sequences of the CDSs associated with individual cDNA clones. With Sanger reads, clones that are not amplified by PCR can be easily detected and then we can sequence them in another run.

Our approach requires that no two full-length cDNA clones in a same library should overlap on the reference genome because of the difficulty in reconstruction of individual full-length cDNA clones overlapping on the reference genome. This requirement is fulfilled by obtaining 5′- (and/or 3′-) Sanger reads to detect overlapping clones by their alignments with the reference genome, making it possible to create a set of “non-overlapping” full-length cDNA clones. If sequencing centers have full-length cDNA clones from a variety of distantly related species, they can distribute them over flowcell lanes to increase throughput while keeping the accuracy, because clones from distantly related species do not interfere much with one another.

Second-generation sequencers perform full-length cDNA sequencing an order of magnitude cheaper and faster than conventional full-length cDNA sequencing using the Sanger method. However, short-read sequencers have higher sequencing error rates than Sanger sequencers and have yet to be characterized regarding sequencing error patterns, and *de novo* assembly algorithms of short shotgun reads are still under development for general application including *de novo* sequencing of mammalian genome or genes. Our solution alleviates these problems by integrating *de novo* and reference assembly approaches, and reconstructed over 95% of the PCR-amplified full-length cDNA clones perfectly in terms of CDS structure. Moreover, the technique was shown to work well even with relatively low sequence coverage and also with a non-human species.

Future emerging sequencing technologies should further improve this field, but our approach provides biologists with a ready-to-use solution to obtain complete CDS of full-length cDNA clones in a less expensive way, providing reliable gene annotation for newly analyzed genomes and associated cDNA clone materials for further wet-lab experiments.

## Methods

### Aligning *de novo* contigs against the reference genome

Contigs produced by an external *de novo* assembler (*de novo* contigs) were aligned to the reference genome using BLAT with default parameters. The output of BLAT was then filtered by a custom Perl script in MuSICA 2 package. For each contig, the alignment with the best score was retained and the others were discarded. If more than one alignment tied in score, they were all discarded.

The reference genome is provided by user. As reference genomes, we used the latest (hg18) human genome assembly [2] downloaded from the University of California, Santa Cruz (UCSC) genome browser ftp site, and the *Toxoplasma* genome from ToxoDB [51] release 4.2.

### Merging overlapping contigs

The BLAT alignments of *de novo* contigs against the reference genome contained erroneous alignments. MuSICA 2 modified those alignments before contig merge.

Manual examination showed that most of those erroneous alignments had very short initial/terminal exons distant from the other exons. Those short exons were aligned to incorrect regions on the genome due to sequencing errors and/or polymorphisms. To eliminate those erroneous exons, initial/terminal exons shorter than 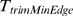 bp (12 bp by default) and distant more than 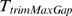 bp (5 bp by default) from the adjacent exon were eliminated. An initial/terminal exon longer than 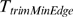 bp was likely to be true even when it was distant from the adjacent exon, while an initial/terminal exon nearer to the adjacent exon than 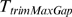 bp might reflect sequencing errors or polymorphisms near the exon-intron boundary. These threshold values were determined empirically, and therefore a slight change would result in a better assembly when the target species or expected polymorphism level differ very much from those for the tested cases.

After alignment improvement, overlapping contigs were merged. As well as two contigs that share at least one base pair on the genome, two contigs aligned exactly next to each other on the genome (often referred to as two contigs with “a zero-base gap”) are merged.

### Aligning raw shotgun reads against the exon gap candidates

To test whether exon gap candidates could be filled as an exon, raw shotgun reads were aligned against them using BLAT and a custom Perl script. To avoid end-effect, the target regions are extended into both sides (i.e., 5′- and 3′-direction) by 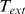 bp, which is by default 50 bp. MuSICA 2 assumes that the read length is shorter than 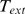 by at least three base pairs; users should set larger value for 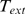 when the read length becomes longer.

Since BLAT was not designed to align short reads, the parameter was carefully chosen so as not to miss alignments within three mismatches or two indels. The actual parameter given to BLAT was “−tileSize = 12 −stepSize = 6 −minMatch = 1 −oneOff = 1 −repMatch = 3000 −minScore = 10.” Alignments with more than three discrepancies (mismatches/indels) were discarded by a custom Perl script in MuSICA 2. Note that most alignments with two or three discrepancies (mismatches/indels) were found but some might be missed due to the algorithmic design of BLAT (Table S5).

The alignment with the best score was kept for each raw read, and the others are discarded. If several alignments tied in score, they were kept but labeled as repetitive.

### Splice-alignment of shotgun reads against the intron gap candidates

To test whether intron gap candidates could be filled, raw shotgun reads were splice-aligned against genomic regions near exon-intron boundaries, which we will refer to as *exon-intron boundary candidate regions*. An exon-intron boundary candidate region was defined as a 2 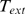-bp (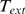 = 50 bp by default) region whose center was the boundary of contigs after exon filling. MuSICA 2 tried to find splice-alignments of reads in which a part of a read was aligned to some exon-intron boundary candidate region and the other part of the read was aligned to the next exon-intron boundary candidate region.

Although BLAT was designed to perform spliced alignments, the chaining algorithm and gap scoring scheme are not appropriate for our purpose. Instead of using BLAT directly, we developed a custom Perl script to perform the splice-alignment. We split every raw read into two parts at several positions, and aligned them separately using BLAT with a permissive parameter set as follows: “−tileSize = 10 −stepSize = 2 −minMatch = 1 −oneOff = 1 −minScore = 10 −repMatch = 1000000.” There are 35 positions at which to split 36-bp read into two parts, and we tried every case except those in which either part of the split read was shorter than 13 bp. Alignment results were then combined and filtered by the custom Perl script. First, up to one mismatch for each part of a read was allowed; other alignments were discarded. Next, *valid* pairs of alignments were kept and the others were discarded. A pair of alignments for a read was *valid* if and only if the 5′-part of the read (or the reverse-complemented read) was aligned to some exon-intron boundary candidate region and the remaining 3′-part to the next exon-intron boundary candidate region. If more than one pair of alignments remained, they were discarded. Since we observed false-positive alignments frequently for a certain class of reads, the following filter was applied to discard them: (a) when a read or its reverse complement contained a stretch of poly-A longer than 12 bp, it was discarded; (b) when the ratio of “A” in a read or its reverse complement exceeded 50%, it was discarded.

### PCR amplification of cDNA and subsequent shotgun sequencing

From the glycerol stocks of full-length cDNAs, cDNA inserts, which were cloned to the DraIII site of the pME18S (GenBank acc. no. AB009864), were amplified by colony-PCR using following materials: one drop (<1µl) of the glycerol stock in 4.93 µl of dH_2_O with 1.0 µl of 10× ExTaq Buffer, 0.9 µl of the four dNTPs at 2.5 mM each, 1.56 µl of each colony-PCR primer (5′- TCAGTGGATGTTGCCTTTAC -3′ and 5′-TGTGGGAGGTTTTTTCTCTA -3′) and 0.05 µl of ExTaq DNA polymerase (TaKaRa); the PCR condition was 30 cycles at 95°C, 15 sec; 55°C , 15 sec; 72°C, 4 min. The PCR-amplified fragments were mixed in equal aqueous volume and fragmented. The fragmentation and subsequent adaptor ligation for the Illumina GA sequencing was as described in the manufacturer's manual (ver 2.3 for sample preparation, ver 3 for sequencing). The adaptor-ligated cDNA fragments were size-fractionated by 12% PAGE, and the fraction of 150–200 bp was recovered. The quality and quantity of the cDNAs were assessed using BioAnalyzer (Agilent). One nano-gram of the obtained cDNA was used for the cluster generation, after which sequence reactions followed according to the standard protocol of Illumina GA. Solexa pipeline version 0.2.2.5 was used for image analysis, cluster detection, base-calling and Quality Value (QV) prediction.

### Computational Performance

MuSICA 2 was run on a Linux cluster with Sun Grid Engine. The whole computation including Velvet assembly for library 1+2 took about 50 minutes using 8 nodes, each of which was dual dual-core (4 cores/node) Opteron 2.6GHz with 16GB of memory. The storage was shared by Network File System.

### Software and Data Availability

The source code of MuSICA 2 and related raw data are freely available at http://musica.gi.k.u-tokyo.ac.jp/

Short reads are deposited into NCBI Short Read Archive (acc. no. SRA001156), and they are also available at the site above.

## Supporting Information

Text S1 Supporting information.
(0.09 MB DOC)

Figure S1 A) Library 1. The minimum length was 717 nt, the maximum was 4,206 nt, the average was 2,058 nt, and the median was 1,964 nt. B) Library 3. The minimum length was 683 nt, the maximum was 3,033 nt, the average was 1,563 nt, and the median was 1,436 nt.
(0.16 MB TIF)

Figure S2 The figure shows the statistics (maximum contig length, N50 contig length and the total length of the output contigs) of the *de novo* assembly results for combinations of all read lengths (26 to 36 bp) and all hash lengths (19 to 29 bp) using reads from Library 1. Maximum length, N50 contig length and total length of the output contigs are shown relative to the length of *de novo* contigs. The accuracy of the MuSICA 2 assembly (the ratio of clones assembled consistently with the reference CDS structures) is represented by the red dotted line (left Y-axis) and was highly correlated with the N50 contig size and the maximum contig size. The best result obtained by MuSICA 2 used Velvet with 32-bp reads and hash length of 23 bp, achieving an accuracy of 98.6%, though its N50 contig size (1,691bp) was slightly shorter than the best N50 contig size (1,721 bp).
(0.14 MB TIF)

Figure S3 **Case 1.** Contigs assembled from Illumina GA short reads were often fragmented. The Sanger reads from both ends of the cDNA clones were extended by combining the Sanger read and the overlapping contig. When the Sanger reads from both ends of the cDNA clone were not linked, there remained a gap of unknown size. When extending the Sanger read, the contig consensus sequence was preferred over the Sanger read in the overlapping region. **Case 2.** A single long contig had an overlap with the Sanger reads from both ends of the cDNA clone. The output had no gap, suggesting successful reconstruction.
(0.10 MB TIF)

Figure S4 The x-axis is the nucleotide position from the beginning of the cDNA, while the y-axis represents the number of alignments that covered the position. The two graphs in the left column show the result for the 5′-end, while those in the right column are for the 3′-end. The two graphs in the top row show the range 0–4 kbp, while those in the bottom row display magnified views of the top two graphs. Red lines show the unique sequence coverage, which is the number of unique alignments that covered a specific nucleotide. Blue lines show the repetitive sequence coverage, which is the number of repetitive alignments that covered a specific nucleotide.
(0.24 MB TIF)

Figure S5 The histograms show the clone length distribution of the clones in Library 1 and 1+2, colored according to the classification of the assembly results for individual clones. Clones classified as “not amplified by PCR” and “inconsistent” were widely distributed and did not show any clear tendency, suggesting that clone length was related with neither PCR failure nor assembly accuracy. Longer DNA fragments are generally more difficult to amplify. We performed Mann-Whitney U-test and 2-sample Kormogorov-Smirnov test to identify potential bias toward shorter clones. The p-values were 0.38 (Mann-Whitney test) and 0.80 (Kormogorov-Smirnov test), respectively, suggesting that, if any, such bias was too weak to identify.
(0.14 MB TIF)

Figure S6 The chart shows the number of CDS-consistent and CDS-inconsistent clones assembled by different assemblers. Among the three assemblers, MuSICA 2 performed better than the others as it could reconstruct more consistent sequences with the least number of inconsistent cases. Output clones that were consistent with the reference CDS structure are colored blue, whereas inconsistent clones are colored red.
(0.11 MB TIF)

Figure S7 The diagram shows the accuracy evaluation process for the 780 Toxoplasma gondii full-length cDNA clones in library 3. Of all the 131 PCR-amplified clones with CDS annotation, 97.7% were consistent with the assembled contigs in terms of CDS structure. With regard to unknown sequences, the contigs associated to the clones had an average length of 1,705bp.
(0.07 MB TIF)

Table S1 **Total raw reads:** the number of Illumina reads outputted by the SolexaPipeline software. **Average QV:** the Quality Value averaged over the total raw reads. **Number of reads without Ns:** the number of reads without Ns (undetermined nucleotides). **Number of reads without poly(A)s:** the number of reads after filtering for poly(A/T) and reads with high ratio of A or T. **Number of reads after purity filtering:** the number of reads that passed the purity filtering. *Average QV was measured using the Illumina Quality Value.
(0.03 MB DOC)

Table S2 This table shows the statistics for two gap filling methods. For each type of gap filling, the number of candidate regions, the total length and the number of alignments found are shown. For exon gap candidates, average coverage was the average sequence coverage calculated using the number of short read aligned against the exon candidate regions.
(0.03 MB DOC)

Table S3 See Table 3 in the main text for details. The units are base pairs.
(0.03 MB DOC)

Table S4 See Table 4 in the main text for details. The units are base pairs.
(0.03 MB DOC)

Table S5 For each library, the aligned reads are categorized by read uniqueness and the number of mismatches/indels in the best alignment. The number is on a read-by-read basis; a read that aligned to multiple locations on the genome was counted only once.
(0.05 MB DOC)
