# Supplementary material for: Cost-Effective Sequencing of Full-Length cDNA Clones Powered by a De Novo-Reference Hybrid Assembly
Source: PLoS One. 2010 May 7;5(5):e10517. doi: 10.1371/journal.pone.0010517 (PMC2866332; doi:10.1371/journal.pone.0010517)
Supplement: Text S1 — Supporting information. (0.09 MB DOC) [file pone.0010517.s001.doc]

**Supporting Information**

***De novo* assembly of short reads**

If it were possible to assemble the shotgun reads obtained from the cDNA libraries *de novo*, it would not be necessary to use reference genomes. Therefore, we first tried to test existing *de novo* assembly algorithms for short reads, such as SSAKE [1], VCAKE [2], SHARCGS [3], Velvet [4], and Edena [5], to study feasibility, and found that existing *de novo* assembly algorithms needed further improvements for multi-clone full-length cDNA shotgun sequencing.

Sequencing errors and repetitive sequences in the cDNA sequences often produce more than one choice of extending the contigs, thereby terminating contig extensions. As a result, the assembly often ends up too short contigs, far from reconstructing entire cDNA sequences. Below is a list of the attempts we made for *de novo* assembly of the shotgun reads of the cDNA clones. All of the tests below were performed only with library 1, which we believe is the best data set of the two human libraries for *de novo* assembly because it had the lowest multiplicity of cDNA clones, and thus had the highest sequence coverage. We did not use SSAKE and VCAKE, as recent *de novo* assembly papers have shown that these two algorithms yield significantly shorter contigs for real data [4-6]. We did not use ALLPATHS [6] either, because its design targets paired-end shotgun reads, which we did not have.

To make a fair comparison between the results of *de novo* assemblers and that of MuSICA 2, we also used the Sanger reads from the 5’- and 3’-end of the cDNA clones to improve the output of *de novo* assemblers according to the procedure in what follows (Fig. S3). The correspondence between individual clones and assembled contigs were identified by aligning the Sanger reads against the contigs. Ideally, each output contig has exactly one corresponding cDNA clone. However, the output contigs were often fragmented. We searched overlaps between the Sanger reads and the output contigs to create as long contigs as possible. We required for an overlap at least 30 bp to be aligned to the Sanger read with a match ratio of more than 90%. For each Sanger read, the contig that aligned to the farthest position from the 5'-end of the Sanger read was used to extend the Sanger read because the strategy is most effective in contig extension. When a contig has overlaps with the Sanger reads from both ends of the full-length cDNA clone at once, we could expect the assembled contig to be the complete sequence of that clone. When no such contig was found, the output cDNA for that clone was the two extended contigs and a gap between them, which indicated partial reconstruction. Contigs with no overlaps with the Sanger reads were discarded, as they were often false positives, or redundant short contigs that were actually a part of another long contig. In regions where a contig and some Sanger read overlapped, we preferred nucleotide sequences from the contig for output because nucleotide sequences from the tail part of one-pass Sanger reads were expected to be of lower quality than the contig assembled from shotgun reads of high redundancy.

To find the best combination of parameters, various combinations of parameters were tried and the best result was taken. The actual parameters were described in the following sections. We had to use the reference genome to evaluate the result of *de novo* assemblers, and thus the optimization of the results depended on the use of the reference genome, implying that the accuracy of Velvet and Edena shown here were potentially overestimated.

To evaluate the assemblies, we aligned the assembled contigs with the reference genome to determine their exon-intron structures using BLAT. The output full-length cDNA sequences might not always align perfectly to the reference genome, and indeed we observed indels as well as mismatches. Deletions equals to or shorter than 5 bp were treated as polymorphisms between the reference genome and the full-length cDNA clone, whereas longer deletions were considered as introns. More precise numbers of mismatches, insertions and deletions obtained in the assembly results are shown in Table 3 and Table 4.

Fig. S6 shows the overall results with three approaches, MuSICA 2, "Velvet + Sanger reads," and "Edena + Sanger reads." Among the three approaches, MuSICA2 performed the best and "Velvet + Sanger reads" followed. Table S3 and S4 show detailed alignment statistics comparing the hybrid strategy with *de novo* assembly approaches.

***De novo* assembly by Velvet**

We tried Velvet with different read lengths ranging from 26 bp to 36 bp and odd *k* values (size of the hash) ranging from 19 to 29. Velvet achieved its best result when the read length was 36 bp and *k*=23, yielding 424 contigs, with the N50 contig size of 1,721 bp. The accuracy of the assembly output was calculated after the addition of the Sanger reads as mentioned above. Of the 199 reference full-length cDNA clones, 156 clones had at least one exon output. By comparing the assembly result with the reference CDS structures, we found that about 84% (117/139) of the output clones were consistent with the reference CDS structures (Fig. S4). The Velvet version we used was 0.5.07. The execution time of a single run for library 1+2 was about 3 minutes requiring 0.9 GB of memory on a Linux machine with two dual-core Opteron processors (2.6 GHz) and 16 GB of memory.

***De novo* assembly by Edena**

Edena was run with read lengths varying from 26 bp to 36 bp and the cutoff value for overlap sizes ranging from 19 to 29. We found that for library 1 the best result was achieved using 26-bp reads and cutoff value of 21 for the overlap size and nonstrict mode. With this configuration, the N50 contig size was 1,684 bp and the number of contigs generated was 346. Of all reference cDNA clones with at least one associated output contig, 80% (112/140) were consistent with the reference CDS structure (Fig. S4) after the addition of the Sanger reads. The software version we used was 2.1.1. A single run of Edena assembly of library 1+2 took about 14 minutes and required 0.7 GB of memory on the same machine as the experiment with Velvet.

***De novo* assembly by SHARCGS**

We attempted to run SHARCGS version 1.2.8 using all combination of [read length 30, 32, 36 bp], [default, -r 2, -r 3], -l 50. However, even the best run (32 bp, -l 50 –r 2) produced more fragmented contigs (the largest contig: 357 bp; the average length: 72 bp). The best run took 186 hours on a Linux machine with an eight-way single-core Opteron 3.0 GHz processor and 128 GB memory, whiｘch was faster and had more memory than that used in the original paper [3]. Note that assembly runs that did not seem to finish in a month were discarded.

**Creating the CDS structures for the 200 reference full-length cDNA clones**

To create a set of reference CDS structures for the 200 full-length cDNA clones in library 1, we aligned them against the human genome using BLAT [7]. The parameters were all default. We required that more than 50% of an end sequence was covered by the alignment; otherwise, it was discarded. When multiple hits were found, a pair of end sequences that aligned on the same target sequence (i.e., scaffold or chromosome) with the best alignment score was chosen. The alignment score of the paired sequences (i.e., Sanger reads from both ends of the clones) was the sum of the alignment scores of the paired sequences. Sometimes we found that two hits tied in their alignment score. Further investigation revealed that one of them was always located on chr_random or chr_Y and the other was on the same (non-random) chromosome or chr_X, respectively. We speculate that they are artificial duplicates due to unmapped clones or the pseudo-autosome region of chromosome Y, both of which may contain redundant sequences. The fact that the observed mismatch patterns in the alignments were all the same supported our speculation. Those putative duplicates were eliminated, after which at most one alignment gave the best score for each clone. In addition, AK000357 in library 1 was chimeric when we assumed that the human genome sequence (hg18) was correct. AK000357 was not amplified by PCR; therefore, it was excluded from further analysis. For library 3, we found more possibly chimeric clones because more clones had alignments to different chromosomes, but we did not simply discard these clones because the strain used for genome sequencing and the strain used for cDNA collection were different, so that more variations including structural variations (or misassemblies) were expected than in the human sequences. Assuming that less aligned sequences are of lower quality or repetitive, we used one end sequence with the better alignment score and the other end sequence was discarded; such clones were treated as if the Sanger read from one end of the cDNA clone was missing. This heuristic aimed at yielding partial assembly results even in the presence of extensive variations including insertion of repetitive elements, misassemblies or structural variations, while keeping the algorithmic design simple.

Due to sequencing errors or polymorphisms, the BLAT alignment did not always start exactly from the very first base of the full-length cDNA clone insert. In such cases, we assumed that the remaining bases could be aligned with no gap to estimate the transcription start site. For example, when the alignment started from the 4^th^ position of the 5’-end sequence (without the vector sequence), the transcription start site was 3 bp before the alignment start position on the genome.

The CDS annotations downloaded from GenBank were then projected onto the exon-intron structure produced by the above procedure. The projected CDSs were used as the reference CDS structures.

**PCR success rate had no statistically significant correlation with clone lengths**

We used PCR amplification to prepare the shotgun templates. Longer DNA fragments are generally more difficult to amplify. Here arose a question whether longer full-length cDNA clones were underrepresented in the set of the PCR-amplified clones.

Fig. S5 shows the clone length distribution categorized by PCR success/failure, in which no apparent correlation could be seen. Next we performed Mann-Whitney U-test and 2-sample Kormogorov-Smirnov test to identify potential bias toward shorter clones. The p-values were 0.38 (Mann-Whitney test) and 0.80 (Kormogorov-Smirnov test), respectively, suggesting that, if any, such bias was too weak to identify.

**The ends of cDNA clones were less represented by shotgun reads**

A uniform distribution of shotgun fragments is the most fundamental basis on which the shotgun sequencing approach relies; however, whether nebulized shotgun fragments are actually distributed uniformly across the target sequence remained unknown.

We observed that the initial or terminal exons tended to have lower sequence coverage. To illustrate this, we calculated the sequence coverage of each nucleotide for the 200 reference cDNAs whose complete nucleotide sequences were determined by the Sanger method (Library 1). The sequence coverage distribution for the reference cDNA clones varied widely due to different efficiency in PCR amplification. Nevertheless, when the sequence coverage of each nucleotide position from the beginning of the clones were summed, there was a clear tendency for both ends of the PCR amplicons to be less represented in the shotgun libraries (Fig. S4). The obtained shotgun reads for library 1 were aligned against the 200 reference cDNAs using BLAT with the same option used in the other analysis in the main text. The unique (or repetitive) sequence coverage for each nucleotide position from each end of the clone insert cDNAs was then calculated by counting the number of unique (or repetitive) alignments that covered the nucleotide position. As shown in the figure, not as many repetitive alignments were observed, so we focused on the unique sequence coverage. At base position 1, the sequence coverage remained low, at nearly zero, rising almost linearly until the peak around 250 bp, suggesting the existence of an end-effect that caused low representation at both ends of the cDNAs. Because most of the reference clones were longer than 1,000 bp, the observed peak around 250 bp could not be attributed to the length distribution of the reference cDNAs. This is presumably due to the physical nature of nebulization being less effective in transferring their energy to DNA molecules in near-terminal regions. Based on this assumption, the plateau ranging from approximately 600 bp to 1,000 bp was the region of uniform distribution. The subsequent descent, ranging from 1,000 bp to 3,000 bp, was consistent with a drop in the number of cDNAs longer than that length. It is also notable that the sequence coverage distributions with regard to the distance from the 5’- and 3’-end of the clones were almost identical in shape, strongly supporting our hypothesis. If sequence coverage were dependent on the nucleotide composition or other sequence properties, the two figures would have different shapes because the nucleotide compositions were different for the regions near the 3’-end and 5’-end.

**Supporting Figure Legends**

**Figure S1. Length distribution of the 200/152 full-length cDNA sequences finished by the conventional primer-walking method.**

1. Library 1. The minimum length was 717 nt, the maximum was 4,206 nt, the average was 2,058 nt, and the median was 1,964 nt.
2. Library 3. The minimum length was 683 nt, the maximum was 3,033 nt, the average was 1,563 nt, and the median was 1,436 nt.

**Figure S2. Relation between the statistics for *de novo* assembly results and the hybrid assembly accuracy shows that MuSICA 2 performed better with longer initial *de novo* contigs.** The figure shows the statistics (maximum contig length, N50 contig length and the total length of the output contigs) of the *de novo* assembly results for combinations of all read lengths (26 to 36 bp) and all hash lengths (19 to 29 bp) using reads from Library 1. Maximum length, N50 contig length and total length of the output contigs are shown relative to the length of *de novo* contigs. The accuracy of the MuSICA 2 assembly (the ratio of clones assembled consistently with the reference CDS structures) is represented by the red dotted line (left Y-axis) and was highly correlated with the N50 contig size and the maximum contig size. The best result obtained by MuSICA 2 used Velvet with 32-bp reads and hash length of 23 bp, achieving an accuracy of 98.6%, though its N50 contig size (1,691bp) was slightly shorter than the best N50 contig size (1,721 bp).

**Figure S3. Contigs generated by *de novo* assemblers were augmented by Sanger reads from both ends of the clones.**

**Case 1.** Contigs assembled from Illumina GA short reads often were often fragmented. The Sanger reads from both ends of the cDNA clones were extended by combining the Sanger read and the overlapping contig. When the Sanger reads from both ends of the cDNA clone were not linked, there remained a gap of unknown size. When extending the Sanger read, the contig consensus sequence was preferred over the Sanger read in the overlapping region. **Case 2.** A single long contig had an overlap with the Sanger reads from both ends of the cDNA clone. The output had no gap, suggesting successful reconstruction.

**Figure S4. Relationship between sequence coverage and nucleotide position from both ends of the reference cDNAs.**

The x-axis is the nucleotide position from the beginning of the cDNA, while the y-axis represents the number of alignments that covered the position. The two graphs in the left column show the result for the 5'-end, while those in the right column are for the 3'-end. The two graphs in the top row show the range 0–4 kbp, while those in the bottom row display magnified views of the top two graphs. Red lines show the unique sequence coverage, which is the number of unique alignments that covered a specific nucleotide. Blue lines show the repetitive sequence coverage, which is the number of repetitive alignments that covered a specific nucleotide.

**Figure S5. Clone length did not have statistically significant correlation with PCR failure rate.** The histograms show the clone length distribution of the clones in Library 1 and 1+2. X-axis is the length of the clones, while the Y-axis is the number of clones. The clones were colored according to the classification of the assembly results for individual clones, and the areas of different colors reflect the proportion of different assembly result classification. Clones classified as “not amplified by PCR” and “inconsistent” were widely distributed and did not show any clear tendency, suggesting that clone length was related with neither PCR failure nor assembly accuracy.

**Figure S6. Comparison of assembly results of Library 1.**

The chart shows the number of CDS-consistent and CDS-inconsistent clones assembled by different assemblers. Among the three assemblers, MuSICA 2 performed better than the others as it could reconstruct more consistent sequences with the least number of inconsistent cases. Output clones that were consistent with the reference CDS structure are colored blue, whereas inconsistent clones are colored red.

**Figure S7. The clone fate diagram for library 3.**

The diagram shows the accuracy evaluation process for the 780 *Toxoplasma gondii* full-length cDNA clones in library 3. Of all the 131 PCR-amplified clones with CDS annotation, 97.7% were consistent with the assembled contigs in terms of CDS structure. With regard to unknown sequences, the contigs associated to the clones had an average length of 1,705bp.

References

1. Warren RL, Sutton GG, Jones SJ, Holt RA (2007) Assembling millions of short DNA sequences using SSAKE. Bioinformatics 23: 500-501.

2. Jeck WR, Reinhardt JA, Baltrus DA, Hickenbotham MT, Magrini V, et al. (2007) Extending assembly of short DNA sequences to handle error. Bioinformatics 23: 2942-2944.

3. Dohm JC, Lottaz C, Borodina T, Himmelbauer H (2007) SHARCGS, a fast and highly accurate short-read assembly algorithm for de novo genomic sequencing. Genome Res 17: 1697-1706.

4. Zerbino DR, Birney E (2008) Velvet: algorithms for de novo short read assembly using de Bruijn graphs. Genome Res 18: 821-829.

5. Hernandez D, Francois P, Farinelli L, Osteras M, Schrenzel J (2008) De novo bacterial genome sequencing: millions of very short reads assembled on a desktop computer. Genome Res 18: 802-809.

6. Butler J, MacCallum I, Kleber M, Shlyakhter IA, Belmonte MK, et al. (2008) ALLPATHS: de novo assembly of whole-genome shotgun microreads. Genome Res 18: 810-820.

7. Kent WJ (2002) BLAT–the BLAST-like alignment tool. Genome Res 12: 656-664.
