# Supplementary figures and images for: Cost-Effective Sequencing of Full-Length cDNA Clones Powered by a De Novo-Reference Hybrid Assembly
Source: PLoS One. 2010 May 7;5(5):e10517. doi: 10.1371/journal.pone.0010517 (PMC2866332; doi:10.1371/journal.pone.0010517)

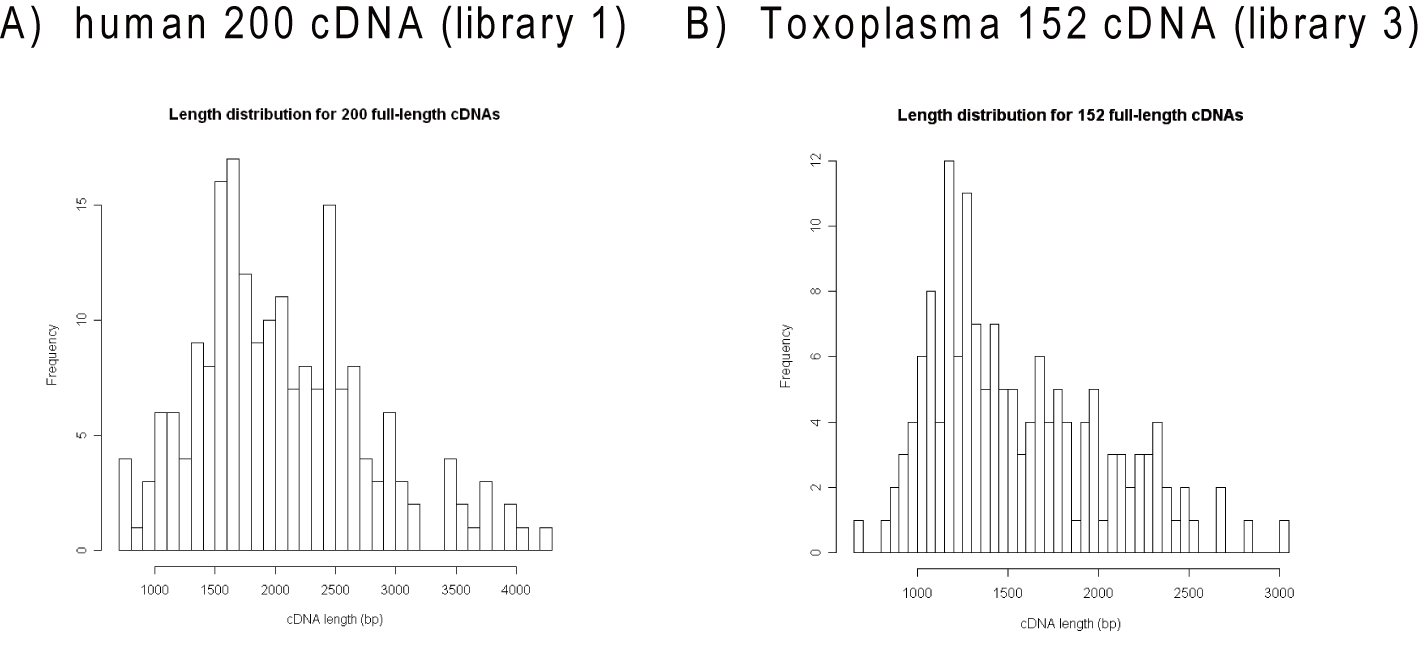

Supplement: Figure S1 — A) Library 1. The minimum length was 717 nt, the maximum was 4,206 nt, the average was 2,058 nt, and the median was 1,964 nt. B) Library 3. The minimum length was 683 nt, the maximum was 3,033 nt, the average was 1,563 nt, and the median was 1,436 nt. (0.16 MB TIF) [file pone.0010517.s002.tif]

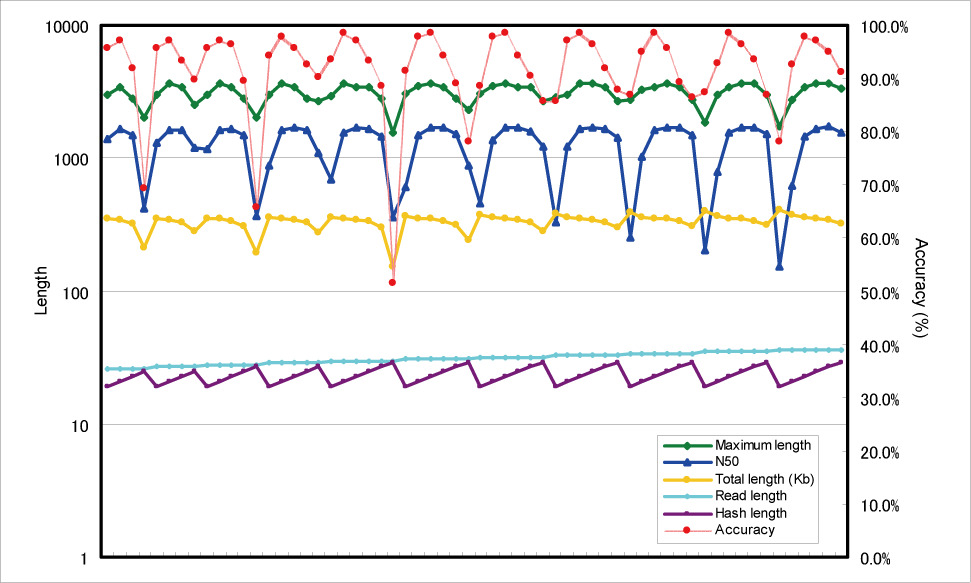

Supplement: Figure S2 — The figure shows the statistics (maximum contig length, N50 contig length and the total length of the output contigs) of the de novo assembly results for combinations of all read lengths (26 to 36 bp) and all hash lengths (19 to 29 bp) using reads from Library 1. Maximum length, N50 contig length and total length of the output contigs are shown relative to the length of de novo contigs. The accuracy of the MuSICA 2 assembly (the ratio of clones assembled consistently with the reference CDS structures) is represented by the red dotted line (left Y-axis) and was highly correlated with the N50 contig size and the maximum contig size. The best result obtained by MuSICA 2 used Velvet with 32-bp reads and hash length of 23 bp, achieving an accuracy of 98.6%, though its N50 contig size (1,691bp) was slightly shorter than the best N50 contig size (1,721 bp). (0.14 MB TIF) [file pone.0010517.s003.tif]

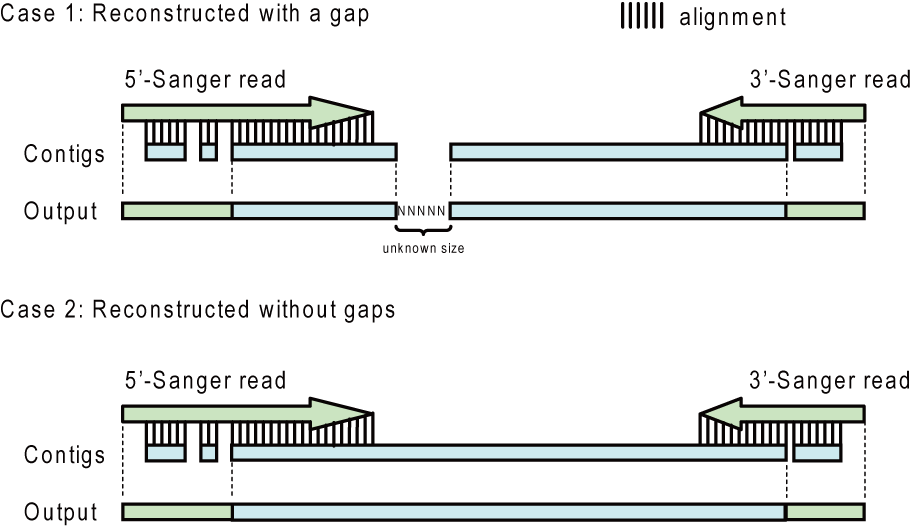

Supplement: Figure S3 — Case 1. Contigs assembled from Illumina GA short reads were often fragmented. The Sanger reads from both ends of the cDNA clones were extended by combining the Sanger read and the overlapping contig. When the Sanger reads from both ends of the cDNA clone were not linked, there remained a gap of unknown size. When extending the Sanger read, the contig consensus sequence was preferred over the Sanger read in the overlapping region. Case 2. A single long contig had an overlap with the Sanger reads from both ends of the cDNA clone. The output had no gap, suggesting successful reconstruction. (0.10 MB TIF) [file pone.0010517.s004.tif]

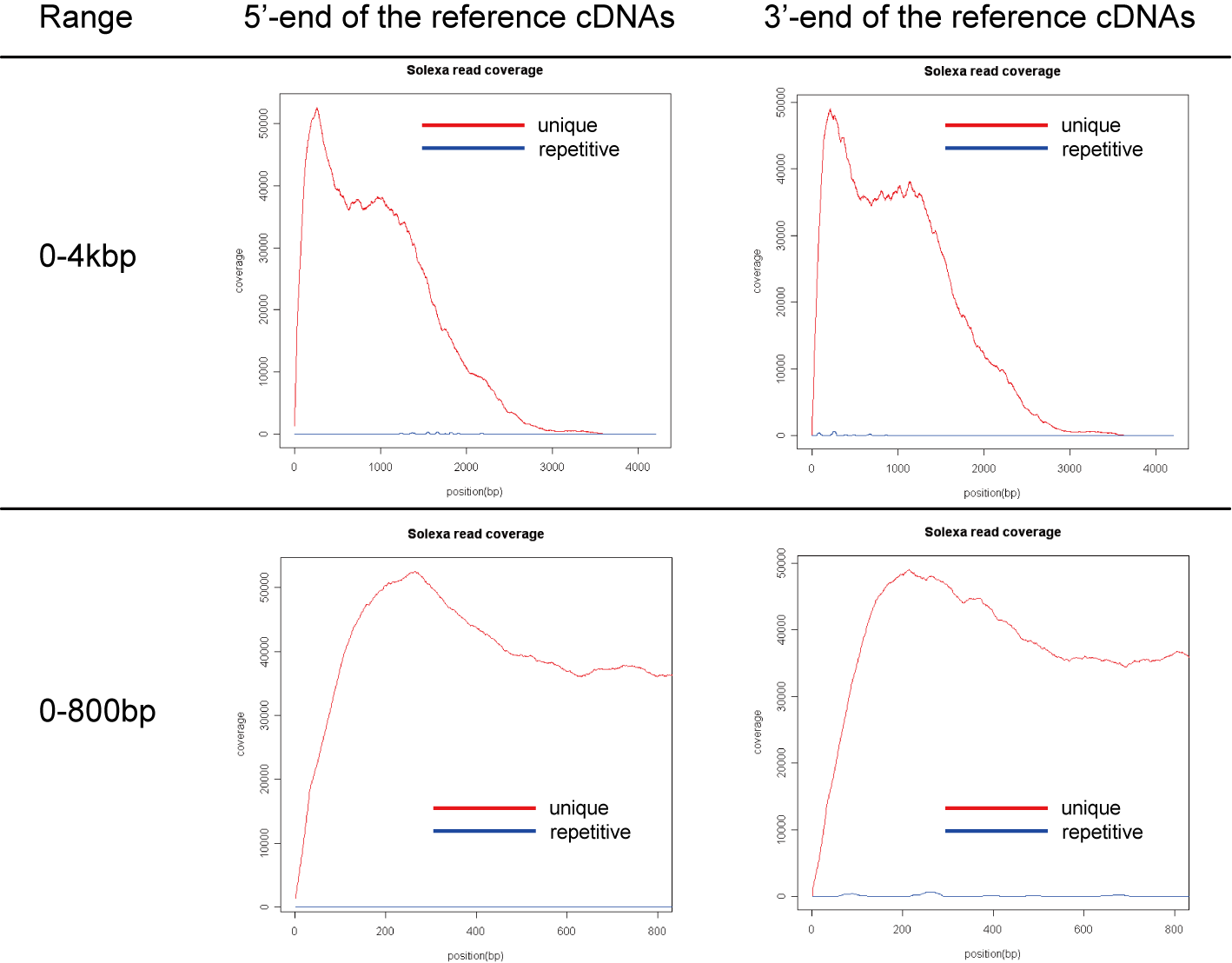

Supplement: Figure S4 — The x-axis is the nucleotide position from the beginning of the cDNA, while the y-axis represents the number of alignments that covered the position. The two graphs in the left column show the result for the 5′-end, while those in the right column are for the 3′-end. The two graphs in the top row show the range 0–4 kbp, while those in the bottom row display magnified views of the top two graphs. Red lines show the unique sequence coverage, which is the number of unique alignments that covered a specific nucleotide. Blue lines show the repetitive sequence coverage, which is the number of repetitive alignments that covered a specific nucleotide. (0.24 MB TIF) [file pone.0010517.s005.tif]

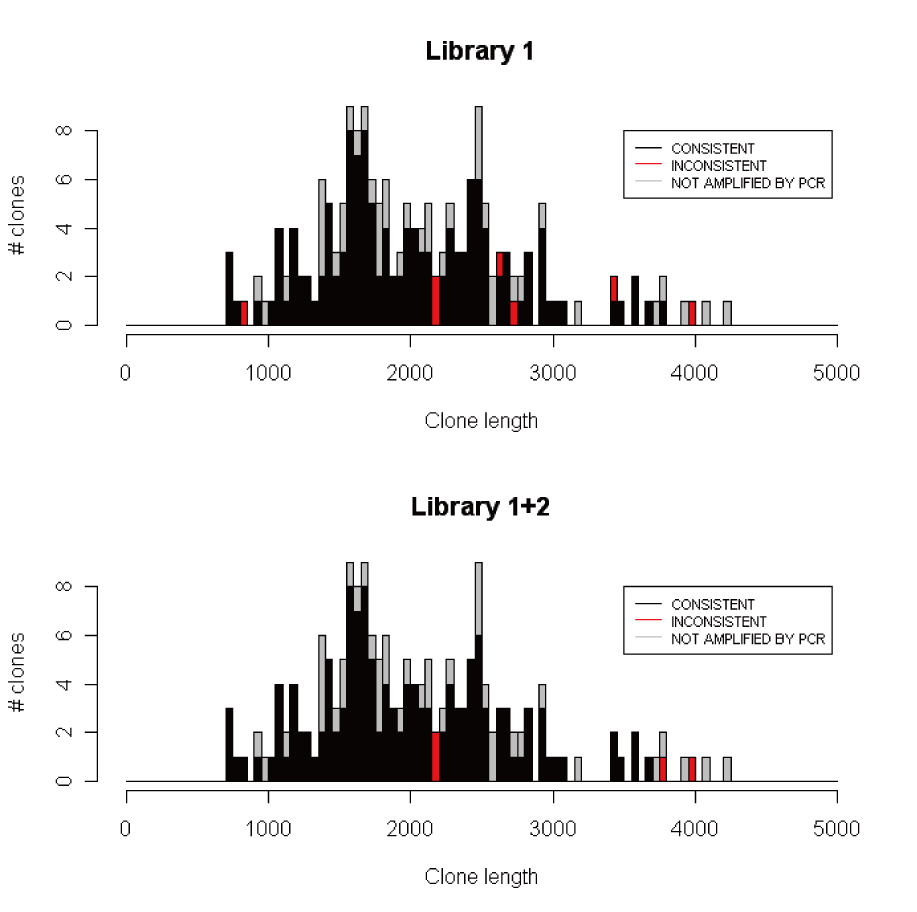

Supplement: Figure S5 — The histograms show the clone length distribution of the clones in Library 1 and 1+2, colored according to the classification of the assembly results for individual clones. Clones classified as “not amplified by PCR” and “inconsistent” were widely distributed and did not show any clear tendency, suggesting that clone length was related with neither PCR failure nor assembly accuracy. Longer DNA fragments are generally more difficult to amplify. We performed Mann-Whitney U-test and 2-sample Kormogorov-Smirnov test to identify potential bias toward shorter clones. The p-values were 0.38 (Mann-Whitney test) and 0.80 (Kormogorov-Smirnov test), respectively, suggesting that, if any, such bias was too weak to identify. (0.14 MB TIF) [file pone.0010517.s006.tif]

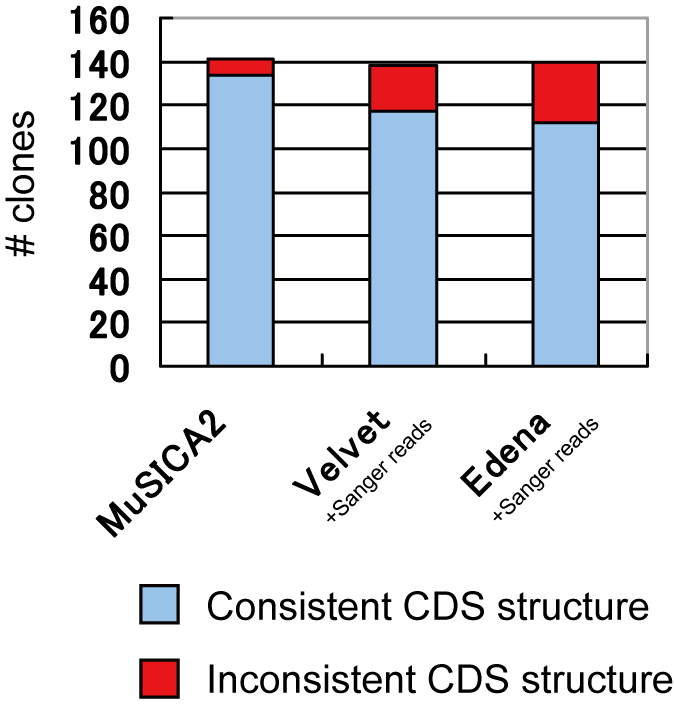

Supplement: Figure S6 — The chart shows the number of CDS-consistent and CDS-inconsistent clones assembled by different assemblers. Among the three assemblers, MuSICA 2 performed better than the others as it could reconstruct more consistent sequences with the least number of inconsistent cases. Output clones that were consistent with the reference CDS structure are colored blue, whereas inconsistent clones are colored red. (0.11 MB TIF) [file pone.0010517.s007.tif]

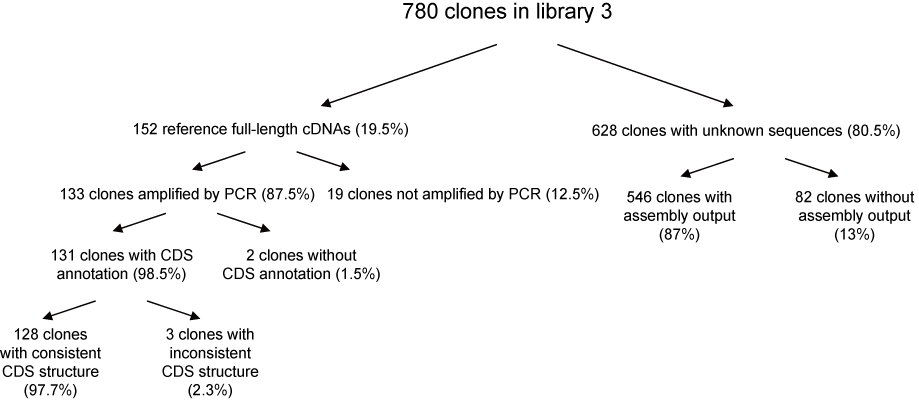

Supplement: Figure S7 — The diagram shows the accuracy evaluation process for the 780 Toxoplasma gondii full-length cDNA clones in library 3. Of all the 131 PCR-amplified clones with CDS annotation, 97.7% were consistent with the assembled contigs in terms of CDS structure. With regard to unknown sequences, the contigs associated to the clones had an average length of 1,705bp. (0.07 MB TIF) [file pone.0010517.s008.tif]
