# Supplementary material for: Cost-Effective Sequencing of Full-Length cDNA Clones Powered by a De Novo-Reference Hybrid Assembly
Source: PLoS One. 2010 May 7;5(5):e10517. doi: 10.1371/journal.pone.0010517 (PMC2866332; doi:10.1371/journal.pone.0010517)
Supplement: Table S1 — Total raw reads: the number of Illumina reads outputted by the SolexaPipeline software. Average QV: the Quality Value averaged over the total raw reads. Number of reads without Ns: the number of reads without Ns (undetermined nucleotides). Number of reads without poly(A)s: the number of reads after filtering for poly(A/T) and reads with high ratio of A or T. Number of reads after purity filtering: the number of reads that passed the purity filtering. *Average QV was measured using the Illumina Quality Value. (0.03 MB DOC) [file pone.0010517.s009.doc]

**Supporting Table 1. Summary of Illumina Genome Analyzer reads.**

|  | Total raw reads | Average QV* | Number of reads w/o Ns | | Number of reads without poly(A)s | | Number of reads after purity filtering | |
| --- | --- | --- | --- | --- | --- | --- | --- | --- |
|  | (A) |  | (B) | (B/A) | (C) | (C/B) | (D) | (D/A) |
| Library 1 | 4,157,115 | 27.88 | 4,045,290 | 97.31% | 2,987,143 | 73.84% | 2,769,069 | 66.61% |
| Library 1 + 2 | 4,608,463 | 27.94 | 4,467,448 | 96.94% | 3,417,441 | 76.50% | 3,141,144 | 68.16% |
| Library 3 | 6,375,052 | 15.77 | 5,406,946 | 84.81% | 4,782,839 | 88.46% | 2,099,122 | 32.93% |

*Average QV was measured using the Illumina Quality Value.

**Total raw reads:** the number of Illumina reads outputted by the SolexaPipeline software. **Average QV:** the Quality Value averaged over the total raw reads. **Number of reads without Ns:** the number of reads without Ns (undetermined nucleotides). **Number of reads without poly(A)s:** the number of reads after filtering for poly(A/T) and reads with high ratio of A or T. **Number of reads after purity filtering:** the number of reads that passed the purity filtering.
