# Supplementary material for: Cost-Effective Sequencing of Full-Length cDNA Clones Powered by a De Novo-Reference Hybrid Assembly
Source: PLoS One. 2010 May 7;5(5):e10517. doi: 10.1371/journal.pone.0010517 (PMC2866332; doi:10.1371/journal.pone.0010517)
Supplement: Table S2 — This table shows the statistics for two gap filling methods. For each type of gap filling, the number of candidate regions, the total length and the number of alignments found are shown. For exon gap candidates, average coverage was the average sequence coverage calculated using the number of short read aligned against the exon candidate regions. (0.03 MB DOC) [file pone.0010517.s010.doc]

**Supporting Table 2. Gap filling statistics.**

|  | Exon gap candidates | | | | Intron gap candidates | | |
| --- | --- | --- | --- | --- | --- | --- | --- |
|  | Number of the exon gap candidate regions | Total length of the exon gap candidate regions (bp) | Number of reads aligned to the regions | Average coverage | Number of exon-intron boundary candidate regions | Total length of exon-intron boundary candidate regions (bp) | Number of splice-alignments |
|  | (A) | (B) | (C) | (36*C/B) | (D) | (E) | (F) |
| Library 1 | 90 | 16,300 | 17,570 | 38.8 | 216 | 21,600 | 26,136 |
| Library 1 + 2 | 672 | 135,852 | 53,500 | 14.2 | 1,980 | 197,756 | 90,059 |
| Library 3 | 355 | 91,856 | 26,274 | 10.3 | 776 | 77,525 | 26,541 |

This table shows the statistics for two gap filling methods. For each type of gap filling, the number of candidate regions, the total length and the number of alignments found are shown. For exon gap candidates, average coverage was the average sequence coverage calculated using the number of short read aligned against the exon candidate regions.
