# Supplementary material for: Cost-Effective Sequencing of Full-Length cDNA Clones Powered by a De Novo-Reference Hybrid Assembly
Source: PLoS One. 2010 May 7;5(5):e10517. doi: 10.1371/journal.pone.0010517 (PMC2866332; doi:10.1371/journal.pone.0010517)
Supplement: Table S3 — See Table 3 in the main text for details. The units are base pairs. (0.03 MB DOC) [file pone.0010517.s011.doc]

**Table S3. Base accuracy comparison of MuSICA 2 with *de novo*** assemblies + Sanger reads.

|  | MuSICA2 | Velvet + Sanger reads | Edena + Sanger reads |
| --- | --- | --- | --- |
| Total matches | 315,861 | 287,171 | 275,571 |
| Total mismatches | 71 | 120 | 142 |
| Total insertions | 245 | 1,698 | 15,882 |
| Total deletions | 1,458 | 7,412 | 2,395 |

See Table 3 in the main text for details. The units are base pairs.
