# Supplementary material for: Cost-Effective Sequencing of Full-Length cDNA Clones Powered by a De Novo-Reference Hybrid Assembly
Source: PLoS One. 2010 May 7;5(5):e10517. doi: 10.1371/journal.pone.0010517 (PMC2866332; doi:10.1371/journal.pone.0010517)
Supplement: Table S4 — See Table 4 in the main text for details. The units are base pairs. (0.03 MB DOC) [file pone.0010517.s012.doc]

**Table S4. Base accuracy comparison of MuSICA 2 and *de novo* assemblies for the CDS of the CDS-consistent output full-length cDNA clones**.

|  | MuSICA2 | Velvet + Sanger reads | Edena + Sanger reads |
| --- | --- | --- | --- |
| Total matches | 132,399 | 107,731 | 103,786 |
| Total mismatches | 6 | 2 | 2 |
| Total insertions | 1 | 22 | 73 |
| Total deletions | 0 | 0 | 0 |

See Table 4 in the main text for details. The units are base pairs.
