# Supplementary material for: Cost-Effective Sequencing of Full-Length cDNA Clones Powered by a De Novo-Reference Hybrid Assembly
Source: PLoS One. 2010 May 7;5(5):e10517. doi: 10.1371/journal.pone.0010517 (PMC2866332; doi:10.1371/journal.pone.0010517)
Supplement: Table S5 — For each library, the aligned reads are categorized by read uniqueness and the number of mismatches/indels in the best alignment. The number is on a read-by-read basis; a read that aligned to multiple locations on the genome was counted only once. (0.05 MB DOC) [file pone.0010517.s013.doc]

**Table S5. Alignment statistics of short reads against exon gap candidates.**

|  |  | Library 1 | | Library 1 + 2 | | Library 3 | |
| --- | --- | --- | --- | --- | --- | --- | --- |
|  |  | Number | ratio | Number | ratio | Number | ratio |
| Unique | Full match | 4,558 | 27.23% | 18,869 | 39.62% | 5,510 | 21.87% |
|  | 1 error | 6,007 | 35.89% | 15,561 | 32.68% | 8,852 | 35.14% |
|  | 2 errors | 3,607 | 21.55% | 7,720 | 16.21% | 6,764 | 26.85% |
|  | 3 errors | 2,566 | 15.33% | 5,469 | 11.48% | 4,064 | 16.13% |
|  | Total | 16,738 | 100.00% | 47,619 | 100.00% | 25,190 | 100.00% |
| Repetitive | Full match | 60 | 7.21% | 1,020 | 17.34% | 246 | 22.69% |
|  | 1 error | 220 | 26.44% | 1,729 | 29.40% | 415 | 38.28% |
|  | 2 errors | 257 | 30.89% | 1,709 | 29.06% | 300 | 27.68% |
|  | 3 errors | 295 | 35.46% | 1,423 | 24.20% | 123 | 11.35% |
|  | Total | 832 | 100.00% | 5,881 | 100.00% | 1,084 | 100.00% |

For each library, the aligned reads are categorized by read uniqueness and the number of mismatches/indels in the best alignment. The number is on a read-by-read basis; a read that aligned to multiple locations on the genome was counted only once.
